# Supplementary material for: Does experiencing a traumatic life event increase the risk of intimate partner violence for young women? A cross-sectional analysis and structural equation model of data from the Stepping Stones and Creating Futures intervention in South Africa
Source: BMJ Open. 2022 Apr 28;12(4):e051969. doi: 10.1136/bmjopen-2021-051969 (PMC9058688; doi:10.1136/bmjopen-2021-051969)
Supplement: Author's manuscript [file bmjopen-2021-051969.draft_revisions.pdf]

BMJ Open

BMJ Open is committed to open peer review. As part of this commitment we make the peer review history of every article we publish publicly available.

When an article is published we post the peer reviewers' comments and the authors' responses online. We also post the versions of the paper that were used during peer review. These are the versions that the peer review comments apply to.

The versions of the paper that follow are the versions that were submitted during the peer review process. They are not the versions of record or the final published versions. They should not be cited or distributed as the published version of this manuscript.

BMJ Open is an open access journal and the full, final, typeset and author-corrected version of record of the manuscript is available on our site with no access controls, subscription charges or pay-per-view fees (http://bmjopen.bmj.com).

If you have any questions on BMJ Open's open peer review process please email info.bmjopen@bmj.com

Experiencing traumatic life events increases young women's risk of recent intimate partner violence in South African informal settlements: A cross sectional analysis and structural equation model

Journal:	*BMJ Open*
Manuscript ID	bmjopen-2021-051969
Article Type:	Original research
Date Submitted by the Author:	01-Apr-2021

Complete List of Authors:	Mannell, Jenevieve ; University College London, Institute for Global Health<br>Minckas, Nicole; University College London, Institute for Global Health<br>Burgess, Rochelle; UCL, Institute for Global Health ; University of Johannesburg, Department of Social Work<br>Chirwa, Esnat; South African Medical Research Council, Gender and Health Division ; University of Witwatersrand, School of Public Health<br>Jewkes, Rachel; South African Medical Research Council, Gender and Health Division<br>Gibbs, Andrew; South African Medical Research Council, Gender and Health Division

Keywords:	Public health < INFECTIOUS DISEASES, Anxiety disorders < PSYCHIATRY, Substance misuse < PSYCHIATRY, Epidemiology < TROPICAL MEDICINE

SCHOLARONE™
Manuscripts

I, the Submitting Author has the right to grant and does grant on behalf of all authors of the Work (as defined in the below author licence), an exclusive licence and/or a non-exclusive licence for contributions from authors who are: i) UK Crown employees; ii) where BMJ has agreed a CC-BY licence shall apply, and/or iii) in accordance with the terms applicable for US Federal Government officers or employees acting as part of their official duties; on a worldwide, perpetual, irrevocable, royalty-free basis to BMJ Publishing Group Ltd ("BMJ") its licensees and where the relevant Journal is co-owned by BMJ to the co-owners of the Journal, to publish the Work in this journal and any other BMJ products and to exploit all rights, as set out in our licence.

The Submitting Author accepts and understands that any supply made under these terms is made by BMJ to the Submitting Author unless you are acting as an employee on behalf of your employer or a postgraduate student of an affiliated institution which is paying any applicable article publishing charge ("APC") for Open Access articles. Where the Submitting Author wishes to make the Work available on an Open Access basis (and intends to pay the relevant APC), the terms of reuse of such Open Access shall be governed by a Creative Commons licence – details of these licences and which Creative Commons licence will apply to this Work are set out in our licence referred to above.

Other than as permitted in any relevant BMJ Author's Self Archiving Policies, I confirm this Work has not been accepted for publication elsewhere, is not being considered for publication elsewhere and does not duplicate material already published. I confirm all authors consent to publication of this Work and authorise the granting of this licence.

For peer review only

Full article title:

Experiencing traumatic life events increases young women's risk of recent intimate partner violence in South African informal settlements: A cross sectional analysis and structural equation model

Each author's complete name and institutional affiliation(s):

Jenevieve Mannell, Institute for Global Health, UCL

Nicole Minckas, Institute for Global Health, UCL

Rochelle A Burgess, Institute for Global Health, UCL

Esnat Chirwa, Gender and Health Research Unit, South African Medical Research Council

Rachel Jewkes, Gender and Health Research Unit, South African Medical Research Council

Andrew Gibbs, Gender and Health Research Unit, South African Medical Research Council

Corresponding author (name, address, phone/fax, e-mail): Jenevieve Mannell, Institute for Global Health, UCL, 30 Guilford Street, WC1N 1EH, j.mannell@ucl.ac.uk

For peer review only

Abstract

Background: Violence has severe implications for women's mental health, and experiencing multiple forms of violence can magnify these effects. However, current understandings of whether experiencing traumatic events increases women's risk of IPV are limited.

Methods: Using women's baseline data from a cluster randomised controlled trial of the *Stepping Stones Creating Futures Intervention* in South Africa, we used multivariate logistic regression models to evaluate associations between exposure to traumatic neighbourhood events (including witnessing murder, being robbed or kidnapped, witnessing and experiencing rape) and IPV in the past 12 months. We then used structural equation modelling (SEM) to examine potential pathways.

Results: Exposure to traumatic events was common among the 677 women surveyed. Over 70% had experienced at least one in their lifetime; one quarter (24%) had experienced 3 or more different events. Women exposed to any traumatic event had a 43% increase in the odds of experiencing IPV in comparison to those with no exposure (aOR1.43, P=<0.000). Exposure to non-partner rape is more strongly associated with IPV than any other traumatic experience. Pathways from exposure to traumatic events and non-partner rape to recent IPV experience are mediated by a latent variable of poor mental health. Food insecurity is associated with all forms of traumatic experience, and is also indirectly associated with IPV through views by women that are unsupportive of gender equality.

Conclusions: Women living in South African informal settlements who witness or experience traumatic events are more likely to experience IPV, and this increases when women are exposed to multiple events. Experiencing traumatic events, and non-partner rape in particular,

For peer review only

has negative effects on women's mental health in ways that increase their vulnerability to IPV. IPV prevention interventions need to consider the broader impacts of women's exposure to neighbourhood violence and severe poverty on IPV risk in settings where these are endemic.

Key words: intimate partner violence, non-partner rape, neighbourhood violence, trauma, mental health, South Africa

For peer review only

Strengths and Limitations of this study

- This study was the first, to our knowledge, to assess associations and potential pathways between young women's experiences of traumatic events (e.g. being robbed at gun point, witnessing murder) and the odds that they will experience recent intimate partner violence;
- Structural equation modelling provides insight into how mental health indicators mediate the relationships between young women's exposure to traumatic events and intimate partner violence, which would not have been possible using logistic regression alone;
- Study participants were randomly sampled as part of baseline data collection for a cluster randomised controlled trial and is therefore representative of the young women living in South Africa's informal settlements;
- The study relies on self-reported measures of symptoms of post-traumatic stress disorder (PTSD) and depression rather than a formal diagnosis; the results therefore reflect women's understanding of symptoms of stress and anxiety and not PTSD or depression as defined in the *Diagnostic and Statistical Manual of Mental Disorders (DSM-5)*;
- From the questions women were asked about traumatic events in their lives, we were only able to assess whether women had experienced multiple types of events and not how many times each event may have been experienced by an individual.

For peer review only

Introduction

Experiencing or witnessing violence can have long-term traumatising effects that undermine women's mental health and wellbeing (1–3). Experiencing multiple types of violence, such as child abuse, intimate partner violence (IPV) and/or armed conflict, has been shown to compound these negative health effects (4–8). Our aim in this article is to contribute a better understanding of the cumulative effects of these different forms of violence and how these play out in the complex lives of women living in economically constrained settings (9,10). This responds to calls to explore the intersectional forms of violence at work in women's lives (11), including the simultaneous impacts of a range of structural violence(s), such as poverty, racism xenophobia and exclusion (12), and their negative mental health consequences (13).

Many women live in dangerous environments where the potential of experiencing community-based violence is extremely high, and studies suggest that this is associated with IPV experience. For instance, women living in settings either experiencing or that have recently experienced armed conflict are at a significantly higher risk of also experiencing IPV (7,14,15). Investigating IPV in Liberia, Kelly and colleagues find that women living in districts experiencing conflict were almost three times as likely to experience IPV compared to individuals in districts without conflict, even four years after peace was declared (16). Studies of women's experiences during armed conflict point to potential pathways through which an experience of war and conflict increases IPV. This includes poor mental health, which increases women's vulnerability to IPV as well as magnifying the negative outcomes of violence (17), and the role of stress and other symptoms of PTSD in increasing men's use of violence in the household (18–21). These risk factors for IPV are often linked theoretically to the gender inequalities that drive violence against women and girls, which are magnified by the highly stressful experiences of people affected by armed conflict (22,23).

For peer review only

Far less in known about the impact of violence in settings which are not conflict or post-conflict settings, but where exposure to traumatic events – such as being robbed at knifepoint, witnessing a murder or experiencing rape – are incredibly common, and the association of these experiences to IPV. Informal settlements in South Africa have a far higher incidence of traumatic events than in the rest of the country, and than many other countries around the world (24,25). In a study of men's exposure to traumatic life events in informal settlements in South Africa, witnessing or experiencing violent events was common with nearly a quarter of men having witnessed the murder of a family or friend (26). In these same informal settlements, two-thirds (65.2%) of women are estimated to have experienced IPV (27). The frequency with which people experience violence in informal settlements contributes to its normalisation, which is rooted in longstanding economic, racial and gender inequalities in South African society (28). This normalisation of violence in turn impacts mental health by upholding toxic masculinities that position strong men as desirable, and delegitimising the stress and fear women feel when experiencing acts of violence (29,30).

To further understand intersections between different forms of violence in women's lives, we investigated the association between women's lifetime exposure to traumatic events and their recent experiences of IPV (past 12 months). We set out to test three main hypotheses: (1) women who experience traumatic events have a higher risk of experiencing IPV, (2) there is a dose relationship between exposure to traumatic events and IPV (e.g. women who experience more traumatic events are more likely to experience IPV), (3) the relationship between exposure to traumatic events and IPV experiences is mediated by poor mental health.

Methods

Design

For peer review only

Our analysis draws on women's data collected as part of the baseline for Stepping Stones and Creating Futures – a cluster randomised controlled trial of an intervention to reduce IPV and HIV vulnerability among young people living in urban informal settlements in South Africa (31). Baseline data were collected between September 2015 and September 2016 in urban informal settlements near Durban (32).

Women included in the Stepping Stones and Creating Futures (SS/CF) trial were 18 to 30 years old, out of school and not currently in formal employment. They were also residents of the informal settlement, although the length of time they had lived in the settlement varied. All participants were recruited by Project Empower, a local NGO with over 20 years of experience working on participatory programming in South Africa's informal settlements. Ethical approval was obtained from the University of Kwa-Zulu Natal and the South African Medical Research Council.

Data collection

The trial included 34 clusters with 19-21 women per cluster. All trial participants completed baseline questionnaires on a mobile phone in English, isiZulu or Xhosa, with the support of same-sex fieldworkers to assist in cases of low literacy. Questionnaires were tailored for women (a separate questionnaire was provided for men) and had logical skips patterns to facilitate survey completion. Questionnaires took between 45 minutes and 1.5 hours to complete. Participants were reimbursed for completing the questionnaire: R100 (~US$7 for those in the intervention arm and R300 (~US$21) in the control arm, because of the differential benefit of being involved in the intervention.

Patient and Public Involvement

The local partner organisation, Project Empower, participated in framing the research questions and planning the SS/CF intervention. They were active collaborators in the study, including quantitative and qualitative data collection and formulating recommendations from

For peer review only

the analysis. Project Empower engaged community networks to ensure permission for community access and provided feedback to community leaders and organisations on the results of the study. The results of the trial have been disseminated to the communities involved. Our analysis will also be shared with young people living in informal settlements as part of a broader commitment to their active participation in interpreting findings about the health issues that affect their lives (33,34).

Measures

IPV was assessed using eight behaviourally specific questions about women's experiences, five about physical IPV and three about sexual IPV, with response options for each item: "never", "once" "few" or "many times". These questions were based on the WHO multi-country study survey on violence against women (35), which has been adapted for South Africa (36). A positive response to any of the questions was coded as having experienced IPV in the past 12 months.

Traumatic experiences, as the exposure variable, was compiled from eight questions about women's past experiences of traumatic events in their lifetime: witnessing the murder of a friend/ family member, witnessing the murder of a stranger, witnessing an armed attack, being robbed at knife or gun point, being kidnapped, witnessing someone being raped, and experiencing non-partner rape. Possible responses to each item were yes or no. This variable was summarised in three ways. First, we generated a binary variable for never/ any experience of trauma. Second, we generated a variable classifying women into those who had (1) never experienced a traumatic event, (2) experienced 1-2 types of events, and (3) experienced 3 or more types of traumatic events (shown in *Table 1*). Third, we generated a variable that summed all the types of trauma into a score (range 0-7).

Women's mental health was assessed via measures of depressive symptoms, symptoms of post-traumatic stress disorder (PTSD), and alcohol use. For depressive

For peer review only

symptoms, the Centre for Epidemiological Studies Depression Scale (CES-D) was used, which asks 20 questions about depressive symptoms in the past week with responses on a four-point Likert scale ranging from ‘never’ to ‘most or all of the time’ (37). Values associated with the Likert scale were summed to create a CES-D score (range 0–57, α=0.88). Symptoms of PTSD were assessed using the Harvard Trauma Questionnaire (38), which includes 16 questions about feelings in the past week related to traumatic events in the past with responses on a four-point Likert scale ranging from ‘not at all’ to ‘extremely.’ Values were summed to create a PTSD score (range 0–45, α=0.9). Harmful alcohol use was assessed using the Alcohol Use Disorder Identification Test (AUDIT) scale, which asks ten questions about past year alcohol use. Values associated with the Likert scale were summed to create an AUDIT score (range 0–38, α=0.81). Harmful alcohol use was defined as an AUDIT score of eight or more (39).

Other measures included demographic data on age, education level (primary/ secondary), and relationship status (married/ living with a partner, boyfriend, no relationship). Food insecurity was assessed using the Household Hunger Scale (40), which asks how often in the past month there was no food to eat in their household, how often a member of the household had gone to sleep hungry, and how often a member of the household had not eaten for a whole day and night because of no food. Possible responses were on a four-point Likert scale (never, sometimes, often, very often), and summed values were recoded as three categorical variables: little or no hunger/ moderate hunger/ severe hunger. Gender views were assessed using the Gender Equitable Men’s scale adapted for South Africa. This included 20 statements about the role and expectations of men and women with a four-point Likert scale from ‘Strongly disagree’ to ‘Strongly agree.’ Higher scores correspond to views that are less supportive of gender equality (range 2–54, α=0.86).

For peer review only

Analysis

All analyses were performed in STATA 16.1 and considered clustering of the survey data using the SVY command. We first provide descriptive statistics for the sample (Table 1) providing numbers and percentages. Second, we calculated the prevalence of the eight traumatic events asked about in the survey (Table 2), and the various sums for overall trauma experiences.

We then carried out descriptive analyses of the relationship between socio-economic and mental health data and exposure to traumatic events (Table 1). We provide percentages and 95% confidence intervals, or mean and 95%CI as appropriate and tested for significant differences using t-tests for continuous variables and chi-squared tests for categorical variables and report p-values.

We used logistic regression to estimate unadjusted and adjusted associations between exposure to traumatic events and IPV experiences in the past 12 months. We first assessed whether each individual traumatic event was associated with IPV, then whether never/any, then never, 1, 2, 3+. We reported odds ratios, 95%CI and p-values. Adjusted models included the covariates age, education, relationship status, harmful alcohol use, food insecurity and gender views.

Second, we built a structural equation model (SEM) to assess whether there were direct and mediated pathways between lifetime traumatic events and past 12 month IPV experience. To build the SEM, we first identified potential pathways drawing on the theoretical literature available. Given the qualitative difference between being raped oneself and witnessing a traumatic event (such as murder or rape), we constructed a latent variable for non-partner rape and separated this from other experiences of traumatic events. We then regressed each variable included in the draft model to all other variables they were hypothesised to be associated with (i.e. continuous outcome scores for food insecurity,

For peer review only

gender views, depression, PTSD, and harmful alcohol use) (Table 4), and included only those pathways that were significant (P>0.05). Based on a previous analysis of the same dataset (27), we constructed a latent variable for food insecurity by separating questions from the household hunger scale into three distinct variables as part of the model. We then assessed how well our model predicted the sample variance-covariance matrix by examining indices of model fit including the standardized root mean squared residual (SRMR <0.1), root mean squared error of approximation (RMSEA <0.05), comparative fit index (CFI >0.95), and Tucker-Lewis Index (TLI >0.95). We modified the model using the MINDICES command in STATA to identify correlations between error terms with a good trade-off between improved model fit and an increase in degrees of freedom (e.g. modification indices >3.84). We then covaried error terms where we could justify this theoretically.

Results

At baseline, 677 young women were recruited and provided data. Roughly one third of the women (30.4%) had completed secondary education (Table 1). Two-thirds were in a relationship: 63.5% had a boyfriend but were not living with them, and 18.2% were either living with a partner or married. Food insecurity was also common with 50.1% of women living in households experiencing moderate food insecurity, and an additional 31.3% with severe food insecurity. Approximately one fifth of women experienced poor mental health: 21% of women were above the mean of 2.5 on the PTSD scale; 45.2% met the cut-off measure of 20/21 for depression; and 23% of women met the cut-off measure of eight or more for harmful alcohol use.

For peer review only

Table 1: Women's socio-demographic and baseline characteristics, by exposure to traumatic events

	n	*%*	No traumatic experience (n=185) *Mean/% [95% CI]*	1-2 traumatic experiences (n=326) *Mean/% [95% CI]*	3+ traumatic experiences (n=166) *Mean/% [95% CI]*	P-value
**Age**						0.97
18-20	142	21.0%	20.0 [14.8, 26.4]	22.09 [17.9, 26.9]	19.9 [14.5, 26.7]	
21-25	313	46.2%	47.6 [40.4, 54.8]	45.4 [40.0, 50.9]	46.4 [38.9, 54.1]	
26-30	222	32.8%	32.4 [26.0, 39.6]	32.5 [27.6, 37.8]	33.73 [26.9, 41.3]	
**Education**						0.02
Secondary complete	206	30.4%	62.7 [55.7, 69.3]	69.9 [64.8, 74.6]	76.5 [69.5, 82.3]	
Secondary incomplete/ no secondary	471	69.6%	37.3 [30.8, 44.3]	30.1 [25.4, 35.2]	23.5 [17.7, 30.5]	
**Relationship Status**						0.82
Married/ Cohabitating	123	18.2%	18.9 [13.9, 25.2]	17.8 [14.0, 22.3]	18.1 [12.9, 24.7]	
Boyfriend	430	63.5%	62.7 [55.5, 69.4]	65.3 [60.0. 70.3]	60.8 [53.1, 68.0]	
No relationship	124	18.3%	18.4 [13.5, 24.5]	16.87 [13.2, 21.3]	21.1 [15.5, 28.0]	
**Food insecurity**						0.16
Mild (no/little hunger)	126	18.6%	22.7 [17.3, 29.2]	17.8 [14.0, 22.3]	15.7 [10.9, 22.0]	
Moderate	339	50.1%	51.9 [44.6, 59.1]	50.3 [44.9, 55.7]	47.6 [40.1, 55.2]	
Severe	212	31.3%	25.4 [19.6, 32.2]	31.9 [27.0, 37.2]	36.8 [29.8, 44.3]	
**Common mental disorders**						
Depression (score)*	677	NA	17.4 [16.0, 18.8]	22.3 [21.1, 23.4]	23.1 [21.4, 24.8]	
PTSD (score)*	677	NA	1.6 [1.5, 1.7]	1.9 [1.9, 2.0]	2.1 [2.0, 2.2]	
Alcohol abuse (score)*	677	NA	0.1 [0.1, 0.2]	0.2 [0.2, 0.3]	0.3 [0.3, 0.4]	
**Gender attitudes**						
(score)	677	NA	25.2 [23.8, 26.5]	26.0 [25.0, 26.0]	25.4 [23.9, 27.0]	

*p-value <0.001: Levels of significance for continuous variables calculated using t-tests (no trauma experience as comparison)

Lifetime experience of traumatic events was common in the lives of these women (Table 2). Over a third had been robbed at knife or gun point (35.2%), and roughly the same number had witnessed an armed attack (34.6%). A quarter (26.1%) had witnessed the murder of a stranger or someone they know, while almost a fifth (17.6%) had witnessed the murder of a friend or family member. One in ten (10.5%) had witnessed someone being raped, and nearly a third (30.4%) had been raped themselves. Over 70% of women had experienced at least one of these traumatic events, while 24% had experienced three or more.

For peer review only

Table 2: Proportion of women who experienced traumatic events by type of event and frequency

	N	% (95% CI)
**By type of traumatic event**		
Witnessed murder friend	119	17.6 [14.7, 20.5]
Witnessed murder stranger	117	26.1 [22.8, 29.5]
Witnessed armed attack	234	34.6 [31.0, 38.2]
Robbed at gun/ knife point	238	35.2 [31.5, 38.8]
Kidnapped	32	4.7 [3.1, 6.3]
Witnessed rape	71	10.5 [8.2, 12.8]
Experienced non-partner rape	205	30.3 [26.8, 33.8]
**Multiple traumatic events**		
Yes	492	72.7 [69.3, 76.0]
**Number of different traumatic events**		
None	185	27.3 [24.1, 30.8]
1 traumatic event	209	30.87 [27.5, 34.5]
2 traumatic events	117	17.28 [14.6, 20.3]
3 or more traumatic events	166	24.5 [21.4, 27.9]

While there is a significant difference in the number of traumatic events experienced by women who have completed secondary education in comparison to those who have not, there is not a significant difference according to age, relationship status or food insecurity. Gender views are also not significantly associated with an increase in exposure to traumatic events. However, the variables signifying common mental disorders (depression, symptoms of PTSD, and harmful alcohol use) are all significantly associated with an increase in the number of traumatic experiences.

Table 3 reports results from the logistic regression. Non-partner rape is the only traumatic experience that has a significant association with IPV experience in the past year when controlling for age, education, relationship status, food insecurity, harmful alcohol use and gender views. Women exposed to any traumatic event other than experiencing non-partner rape have a 43% increase in the odds of experiencing IPV in the past year in the adjusted analysis, compared to those with no trauma exposure (aOR 1.43). When non-partner rape is included as a traumatic experience in the model, women have an 83% increase in the odds of experience IPV in the past year, compared to those with no trauma exposure (aOR 1.83). There also appears to be a relationship whereby the odds of experiencing IPV increases as women are exposed to one or two traumatic events in comparison to those who did not

For peer review only

experience any such events (1 event, aOR 1.66; 2 events, aOR 2.47). However, this dose relationship between exposure to traumatic events and IPV experience tapers off when more than two traumatic events have been experienced (3+ events, aOR 1.71).

Table 3: Associations between exposure to traumatic events and IPV in the past 12 months

Variable	OR (95% CI)	aOR (95% CI)
**Individual traumatic event**		
Witnessed murder friend	1.23 (0.80,1.88)	1.00 (0.63,1.59)
Witnessed murder stranger	1.02 (0.71,1.47)	0.92 (0.62,1.37)
Witnessed armed attack	**1.40 (1.00,1.97)***	1.34 (0.93,1.92)
Robbed at gun/ knife point	1.37 (0.98,1.92)	1.19 (0.82,1.76)
Kidnapped	1.64 (0.72,3.73)	0.85 (0.36,1.93)
Witnessed rape	**1.96 (1.09,3.51)***	1.58 (0.85,2.92)
Experienced non-partner rape	**2.82 (1.91,4.16)*****	**2.35 (1.54,3.57)*****
**Multiple traumatic events**		
Any trauma (excluding non-partner rape)	**1.61 (1.16,2.23)****	**1.43 (1.00,2.04)***
Any trauma (including non-partner rape)	**2.11 (1.49,2.98)*****	**1.83 (1.26,2.66)****
**Number of different traumatic events**		
1 traumatic event	**1.76 (1.17,2.65)*****	**1.66 (1.08,2.57)***
2 traumatic events	**2.88 (1.74,4.78)*****	**2.47 (1.43,4.27)****
3+ traumatic events	**2.17 (1.39,3.36)*****	**1.71 (1.05,2.78)***

Model adjusted for age, education, relationship status, food insecurity, harmful alcohol use and gender views.

Note: Boldface indicates statistical significance (*p<0.05; **p<0.01; ***p<0.001).

Table 4 presents the direct associations between all variables in preparation for the SEM with significant associations (P<0.05) highlighted in bold. Nearly all associations were significant with the exception of the association between food insecurity and alcohol abuse, and several associations with gender attitudes (including exposure to traumatic events, alcohol abuse, depression and PTSD). These associations were removed from our SEM.

For peer review only

Table 4: Assessing potential pathways of association

	Traumatic events	IPV past 12 months	Non-partner rape	Food insecurity	Alcohol abuse	Depress	PTSD
IPV past 12 mo	**0.052**						
Non-partner rape	**0.687**	**0.028**					
Food insecurity	**0.052**	**0.091**	**0.020**				
Alcohol abuse	**0.040**	**0.356**	**0.021**	0.015			
Depression	**0.017**	**0.185**	**0.010**	**0.053**	**0.163**		
PTSD	**0.035**	**0.211**	**0.011**	**0.454**	**0.203**	**0.779**	
Gender attitudes	0.003	**0.081**	0.002	**0.068**	0.019	0.023	0.039

Associations significant at 95%CI in **bold**

In the SEM, the importance of non-partner rape as a traumatic experience meant that it needed to be separated as a variable from other traumatic events to attain a good model fit. The association between exposure to traumatic events (including the covaried effects of non-partner rape) and IPV in the past 12 months was mediated by indicators of poor mental health, specifically symptoms of depression, PTSD and alcohol abuse, which were all increased by trauma experience and which were directly associated with increased IPV. Increased symptoms of poor mental health were also mediated by increased food insecurity. Food insecurity was also associated with IPV experience mediated by inequitable gender views. The SEM was a good fit with the data (RMSEA 0.046; CFI 0.97; TLI 0.961).

Table 5: Structural equation model

**Parameter Direct effects**	**Standardized coefficients**	**SE**	**z**	**P>|z|**	**95% confidence interval**	
Food insecurity→Trauma	0.11	0.04	2.70	0.007	0.03	0.19
Food insecurity→ Non-partner rape	0.15	0.04	3.58	0.000	0.07	0.24
Food insecurity→Gender views	0.27	0.04	7.01	0.000	0.19	0.34
Trauma→Poor mental health	0.15	0.04	3.36	0.000	0.06	0.23
Non-partner rape→Poor mental health	0.63	0.04	15.43	0.000	0.55	0.71
Poor mental health→IPV	0.68	0.03	20.65	0.000	0.62	0.75
Gender views→IPV	0.09	0.03	2.69	0.007	0.02	0.15

Figure 1: Structural equation model of pathways between exposure to traumatic events and IPV in the past 12 months (standardized coefficients)

Discussion

These results support our hypothesis that women living in informal settlements who experience traumatic events are at a higher risk of experiencing IPV. There was also a large

effect size for those experiencing two or more different traumatic events, in comparison to only one event. This acknowledges how women's exposure to multiple and overlapping violence(s) (including violence outside of the household) impacts their risk of IPV and poor mental health outcomes for women, building on the already well-recognised consideration of the role men's exposure to violence in their perpetration of IPV (14,41).

Both the logistic regression and the SEM highlight the overwhelming importance of non-partner rape as a traumatic event in women's risk of experiencing IPV. Non-partner rape is more strongly associated with IPV experience than any other traumatic event assessed in our analysis. This highlights the importance of non-partner rape in women's risk of experiencing IPV, confirming findings from other studies that have highlighted associations between non-partner rape and an increased risk of IPV (42). This may be explained by the devastating consequences of non-partner rape for women's mental health and the importance of self-blame (43) in increasing their vulnerability to other forms of violence, including IPV.

Our analysis also shows the importance of indicators of poor mental health (including depression, symptoms of PTSD, and harmful alcohol use) in mediating the relationship between experiencing traumatic events and IPV, as shown in the SEM. This confirms findings from other studies of the importance of poor mental health and substance abuse as risk factors for IPV (44,45). While our model shows poor mental health leading to increased IPV risk, the evidence suggests that this relationship can also go in the opposite direction, thus feeding a downward spiral whereby poor mental health leads to IPV, which in turn worsens women's mental health (46,47).

Furthermore, our analysis shows how food insecurity (as a proxy for poverty) is a contributing factor in women's exposure to traumatic events. In the SEM, food insecurity plays a role in increasing both non-partner rape and other traumatic events in women's lives.

For peer review only

Moreover, the association between food insecurity and IPV experience is mediated by gender views unsupportive of women's equality, a pathway corroborated by evidence that food insecurity may be an indicator of women's economic dependence on men with implications for their ability to leave violent relationships (27). The role of food insecurity as a compounding factor in women's exposure to trauma has important implications for thinking about poverty as an additional form of structural violence in women's lives (48), and how this in turn compounds the risk of IPV in women's relationships. This contributes important insights to current understandings of the effects of multiple experiences of trauma or *polyvictimisation* (49–51) by highlighting the need to explore violence happening at a structural as well as an interpersonal level.

Limitations

This study has some limitations. Our data relies on self-reported measures of traumatic events, mental health and IPV. In the case of IPV and traumatic events, questions have been framed around specific acts of violence (hitting, slapping, kicking, etc.) consistent with recognised best practice in the field of violence against women. However, this resulted in a limited number of questions being asked about the types of traumatic events women had experienced and likely did not reflect the full range of events that they potentially experience in their lives. The way in which questions about trauma were asked (e.g. *have you experienced any of this list of possible events?*) means that we were only able to assess whether women had experienced multiple types of events rather than how many times each event may have been experienced by an individual. Moreover, the data does not tell us if the events women experienced were actually traumatising or if they led to a mental health disorder. The survey did not ask about diagnosis and self-reporting mental health measures are limited in their diagnostic potential. The study was also cross-sectional and women were not asked about when the traumatic event occurred in their life. It is therefore possible

For peer review only

(although unlikely) that women's experiences of traumatic events happened more recently than their experience of IPV in the past year.

Conclusions

Our results highlight the need for interventions that comprehensively address multiple forms of violence, with particular attention to non-partner rape, as a means of reducing IPV. In settings with extremely high rates of violence, such as South Africa's informal settlements, women are often exposed to a range of traumatic events, and this has negative consequences for their mental health in ways that put them at increased risk of IPV. As a consequence, IPV interventions in these settings require comprehensive mental health strategies and supports as an integral part of intervention activities. Working through women's trauma is not only an optional add-on to IPV prevention interventions, but an integral resource for reducing women's risk of IPV. However, it is important to remember that attention to mental health also needs to be considered alongside the urgent need for structural interventions that address the contexts of violence and poverty in which these women live.

For peer review only

Acknowledgements

The initial study was funded through the What Works To Prevent Violence? A Global Programme on Violence Against Women and Girls (VAWG) funded by the UK Government's Department for International Development (DFID), and managed by the South African Medical Research Council. However, the views expressed do not necessarily reflect the department's official policies and the funders had no role in study design; collection, management, analysis, and interpretation of data; writing of the report; and the decision to submit the paper for publication. The secondary data analysis performed for this article was supported by UKRI MRC Global Challenge Research Fund MR/T029803/1 and UKRI Future Leaders Fellowship MR/S033629/1. We would also like to sincerely thank all of the participants in the study for their time.

For peer review only

References

1. Caetano R, Cunradi C. Intimate partner violence and depression among Whites, Blacks, and Hispanics. Annals of epidemiology. 2003 Nov;13(10):661–5.

2. Erolin KS, Wieling E, Parra RE. Family violence exposure and associated risk factors for child PTSD in a Mexican sample. Child abuse & neglect. 2014 Jun;38(6):1011–22.

3. Shen AC. Long-term effects of interparental violence and child physical maltreatment experiences on PTSD and behavior problems: a national survey of Taiwanese college students. Child abuse & neglect. 2009 Mar;33(3):148–60.

4. Afifi TO, Taillieu T, Zamorski MA, Turner S, Cheung K, Sareen J. Association of child abuse exposure with suicidal ideation, suicide plans, and suicide attempts in military personnel and the general population in Canada. JAMA Psychiatry. 2016;73(3):229–38.

5. Chiu GR, Lutfey KE, Litman HJ, Link CL, Hall SA, McKinlay JB. Prevalence and overlap of childhood and adult physical, sexual, and emotional abuse: a descriptive analysis of results from the Boston Area Community Health (BACH) survey. Violence and victims. 2013;28(3):381–402.

6. de Jong JT, Komproe IH, Van Ommeren M, El Masri M, Araya M, Khaled N, et al. Lifetime events and posttraumatic stress disorder in 4 postconflict settings. Jama. 2001 Aug 1;286(5):555–62.

7. Gupta J, Falb KL, Carliner H, Hossain M, Kpebo D, Annan J. Associations between Exposure to Intimate Partner Violence, Armed Conflict, and Probable PTSD among Women in Rural Côte d'Ivoire. PLoS One [Internet]. 2014 May 13 [cited 2020 Mar 13];9(5). Available from: https://www.ncbi.nlm.nih.gov/pmc/articles/PMC4019518/

8. Kinyanda E, Weiss HA, Mungherera M, Onyango-Mangen P, Ngabirano E, Kajungu R, et al. Intimate partner violence as seen in post-conflict eastern Uganda: Prevalence, risk factors and mental health consequences Health and human rights of marginalized populations. BMC International Health and Human Rights [Internet]. 2016;16(1). Available from: https://www.scopus.com/inward/record.uri?eid=2-s2.0-84957109647&partnerID=40&md5=baae54ae31d02346e52cb6c08a50a16e

9. Guedes A, Bott S, Garcia-Moreno C, Colombini M. Bridging the gaps: a global review of intersections of violence against women and violence against children. Global health action. 2016;9:31516.

10. Heise L, Fulu E. State of the field of violence against women and girls: what do we know and what are the knowledge gaps. [Internet]. Pretoria: South African Medical Research Council.; 2014. Available from: https://assets.publishing.service.gov.uk/media/57a089b640f0b6497400021a/What_Works_Inception_Report_June_2014_AnnexD_WG1_paper_State_of_the_field.pdf

11. Kazdin AE. Conceptualizing the challenge of reducing interpersonal violence. Psychology of Violence. 2011;1(3):166–87.

12. Galtung J, Fischer D. Violence: Direct, Structural and Cultural. In: Galtung J, Fischer D, editors. Johan Galtung: Pioneer of Peace Research [Internet]. Berlin, Heidelberg:

For peer review only

Springer; 2013 [cited 2021 Mar 15]. p. 35–40. (SpringerBriefs on Pioneers in Science and Practice). Available from: https://doi.org/10.1007/978-3-642-32481-9_3

13. Burgess RA, Fonseca L. Re-thinking recovery in post-conflict settings: Supporting the mental well-being of communities in Colombia. Global Public Health. 2020 Feb 1;15(2):200–19.

14. Annan J, Brier M. The risk of return: Intimate partner violence in Northern Uganda's armed conflict. Social Science & Medicine. 2010 Jan 1;70(1):152–9.

15. Jewkes R, Jama-Shai N, Sikweyiya Y. Enduring impact of conflict on mental health and gender-based violence perpetration in Bougainville, Papua New Guinea: A cross-sectional study. PLOS ONE. 2017 Oct 25;12(10):e0186062.

16. Kelly JTD, Colantuoni E, Robinson C, Decker MR. From the battlefield to the bedroom: a multilevel analysis of the links between political conflict and intimate partner violence in Liberia. BMJ Global Health. 2018 Apr 1;3(2):e000668.

17. Jewkes R, Corboz J, Gibbs A. Trauma exposure and IPV experienced by Afghan women: Analysis of the baseline of a randomised controlled trial. PLOS ONE. 2018 Oct 10;13(10):e0201974.

18. Colson E. War and domestic violence. Cultural survival quarterly. 1995 Spring;35–8.

19. Gibbs A, Said N, Corboz J, Jewkes R. Factors associated with 'honour killing' in Afghanistan and the occupied Palestinian Territories: Two cross-sectional studies. PLoS One [Internet]. 2019 Aug 8 [cited 2020 Mar 10];14(8). Available from: https://www.ncbi.nlm.nih.gov/pmc/articles/PMC6687286/

20. Mannell J, Ahmad L, Ahmad A. Narrative storytelling as mental health support for women experiencing gender-based violence in Afghanistan. Social Science & Medicine. 2018 Oct 1;214:91–8.

21. Stark L, Roberts L, Wheaton W, Acham A, Boothby N, Ager A. Measuring violence against women amidst war and displacement in northern Uganda using the "neighbourhood method." Journal of Epidemiology and Community Health. 2010;64(12):1056–61.

22. Ahmad L, Avoine PA. Misogyny in 'post-war' Afghanistan: the changing frames of sexual and gender-based violence. Journal of Gender Studies. 2016 Jul 28;27(1):86–101.

23. Mannell J, Grewal G, Ahmad L, Ahmad A. A Qualitative Study of Women's Lived Experiences of Conflict and Domestic Violence in Afghanistan. Violence Against Women. 2020 Jul 6;1077801220935191.

24. Kaminer D, Plessis B du, Hardy A, Benjamin A. Exposure to Violence Across Multiple Sites Among Young South African Adolescents. Peace and Conflict: Journal of Peace Psychology. 2013 May;19(2):112–24.

For peer review only

25. Wakefield L, Tait S. Crime and Violence in Formal and Informal Urban Spaces in South Africa Citizen Security Dialogues Dispatches from South Africa: Foreword. Stability: International Journal of Security and Development. 2015 May 5;4(1):Art. 26.

26. Gibbs A, Hatcher A, Jewkes R, Sikweyiya Y, Washington L, Dunkle K, et al. Associations Between Lifetime Traumatic Experiences and HIV-Risk Behaviors Among Young Men Living in Informal Settlements in South Africa: A Cross-Sectional Analysis and Structural Equation Model. J Acquir Immune Defic Syndr. 2019 Jun 1;81(2):193–201.

27. Gibbs A, Jewkes R, Willan S, Washington L. Associations between poverty, mental health and substance use, gender power, and intimate partner violence amongst young (18-30) women and men in urban informal settlements in South Africa: A cross-sectional study and structural equation model. PLOS ONE. 2018 Oct 3;13(10):e0204956.

28. Lamb S, Snodgrass L. Growing up with normalised violence : narratives of South African youth. Commonwealth Youth and Development. 2013 Jan 1;11(1):4–21.

29. Gordon SF, Collins A. “We face rape. We face all things”: Understandings of gender-based violence amongst female students at a South African university. African Safety Promotion: A Journal of Injury and Violence Prevention. 2013;11(2):93–106.

30. Willan S, Ntini N, Gibbs A, Jewkes R. Exploring young women’s constructions of love and strategies to navigate violent relationships in South African informal settlements. Culture, Health & Sexuality. 2019 Jan 11;0(0):1–15.

31. Gibbs A, Washington L, Willan S, Ntini N, Khumalo T, Mbatha N, et al. The Stepping Stones and Creating Futures intervention to prevent intimate partner violence and HIV-risk behaviours in Durban, South Africa: study protocol for a cluster randomized control trial, and baseline characteristics. BMC Public Health. 2017 Apr 20;17(1):336.

32. Gibbs A, Washington L, Abdelatif N, Chirwa E, Willan S, Shai N, et al. Stepping Stones and Creating Futures Intervention to Prevent Intimate Partner Violence Among Young People: Cluster Randomized Controlled Trial. Journal of Adolescent Health. 2020 Mar 1;66(3):323–35.

33. Mannell J, Willan S, Shahmanesh M, Seeley J, Sherr L, Gibbs A. Why interventions to prevent intimate partner violence and HIV have failed young women in southern Africa. Journal of the International AIDS Society. 2019;22(8):e25380.

34. Mannell J, Davis K. Evaluating Complex Health Interventions With Randomized Controlled Trials: How Do We Improve the Use of Qualitative Methods?: Qualitative Health Research [Internet]. 2019 Mar 14 [cited 2019 Mar 15]; Available from: https://journals.sagepub.com/eprint/jEbAMi4r9usX24a8mU4K/full

35. García-Moreno C, Jansen HAFM, Ellsberg M, Heise L, Watts C. WHO multi-country study on women’s health and domestic violence against women: initial results on prevalence, health outcomes and women’s responses. [Internet]. Geneva: World Health Organisation; 2005. Available from: https://www.who.int/reproductivehealth/publications/violence/24159358X/en/

For peer review only

36. Jewkes R, Nduna M, Levin J, Jama N, Dunkle K, Khuzwayo N, et al. A cluster randomized-controlled trial to determine the effectiveness of Stepping Stones in preventing HIV infections and promoting safer sexual behaviour amongst youth in the rural Eastern Cape, South Africa: trial design, methods and baseline findings. Tropical Medicine & International Health. 2006;11(1):3–16.

37. Radloff LS. The CES-D Scale: A Self-Report Depression Scale for Research in the General Population. Applied Psychological Measurement. 1977 Jun 1;1(3):385–401.

38. Mollica RF, Caspi-Yavin Y, Bollini P, Truong T, Tor S, Lavelle J. The Harvard Trauma Questionnaire. Validating a cross-cultural instrument for measuring torture, trauma, and posttraumatic stress disorder in Indochinese refugees. Journal of nervous and mental disease. 1992;180(2):111–6.

39. Saunders JB, Aasland OG, Babor TF, Fuente JRDL, Grant M. Development of the Alcohol Use Disorders Identification Test (AUDIT): WHO Collaborative Project on Early Detection of Persons with Harmful Alcohol Consumption-II. Addiction. 1993;88(6):791–804.

40. Deitchler M, Ballard T, Swindale A, Coates J. Validation of a measure of household hunger for cross-cultural use. In Washington, D.C.: Academy for Educational Development; 2010.

41. Rees S, Thorpe R, Tol W, Fonseca M, Silove D. Testing a cycle of family violence model in conflict-affected, low-income countries: A qualitative study from Timor-Leste. Social Science and Medicine. 2015;130:284–91.

42. Jewkes R, Fulu E, Roselli T, Garcia-Moreno C. Prevalence of and factors associated with non-partner rape perpetration: findings from the UN Multi-country Cross-sectional Study on Men and Violence in Asia and the Pacific. The Lancet Global Health. 2013 Oct 1;1(4):e208–18.

43. Campbell R, Dworkin E, Cabral G. An Ecological Model of the Impact of Sexual Assault On Women's Mental Health. Trauma, Violence, & Abuse. 2009 Jul 1;10(3):225–46.

44. Avanci J, Assis S, Oliveira R. A cross-sectional analysis of women's mental health problems: Examining the association with different types of violence among a sample of Brazilian mothers. BMC Women's Health [Internet]. 2013;13(1). Available from: https://www.scopus.com/inward/record.uri?eid=2-s2.0-84876111818&partnerID=40&md5=1280a3617f5f2bb2b675f852c77b2392

45. Gibbs A, Dunkle K, Ramsoomar L, Willan S, Shai NJ, Chatterji S, et al. New learnings on drivers of men's physical and/or sexual violence against their female partners, and women's experiences of this, and the implications for prevention interventions. Global Health Action. 2020 Dec 31;13(1):1739845.

46. Devries KM, Mak JY, Bacchus LJ, Child JC, Falder G, Petzold M, et al. Intimate Partner Violence and Incident Depressive Symptoms and Suicide Attempts: A Systematic Review of Longitudinal Studies. PLoS Med. 2013 May 7;10(5):e1001439.

For peer review only

47. Machisa MT, Christofides N, Jewkes R. Mental ill health in structural pathways to women's experiences of intimate partner violence. PLOS ONE. 2017 Apr 6;12(4):e0175240.

48. Farmer P. An Anthropology of Structural Violence. Current Anthropology. 2004 Jun 1;45(3):305–25.

49. Finkelhor D, Ormrod RK, Turner HA. Polyvictimization and trauma in a national longitudinal cohort. Development and Psychopathology. 2007 Jan;19(1):149–66.

50. Herrenkohl TI, Sousa C, Tajima EA, Herrenkohl RC, Moylan CA. Intersection of child abuse and children's exposure to domestic violence. Trauma, violence & abuse. 2008 Apr;9(2):84–99.

51. Logie CH, Okumu M, Mwima S, Hakiza R, Irungi KP, Kyambadde P, et al. Social ecological factors associated with experiencing violence among urban refugee and displaced adolescent girls and young women in informal settlements in Kampala, Uganda: a cross-sectional study. Confl Health. 2019 Dec 17;13(1):60.

For peer review only

Contribution Statement

JM and AG conceptualised the paper. The analysis was completed by JM with assistance from EC and AG, who verified the results at multiple time points. AG, RJ and EC were involved in the cluster RCT that provided the baseline data for this analysis. There was substantial intellectual input from RJ , NM and RB during multiple rounds of analysis and drafts of the article. RB also contributed specifically to the interpretation of the analysis of the mental health results.

Data sharing

De-identified data set for this project is available from https://medat.samrc.ac.za/index.php/catalog/23 managed by the South African Medical Research Council.

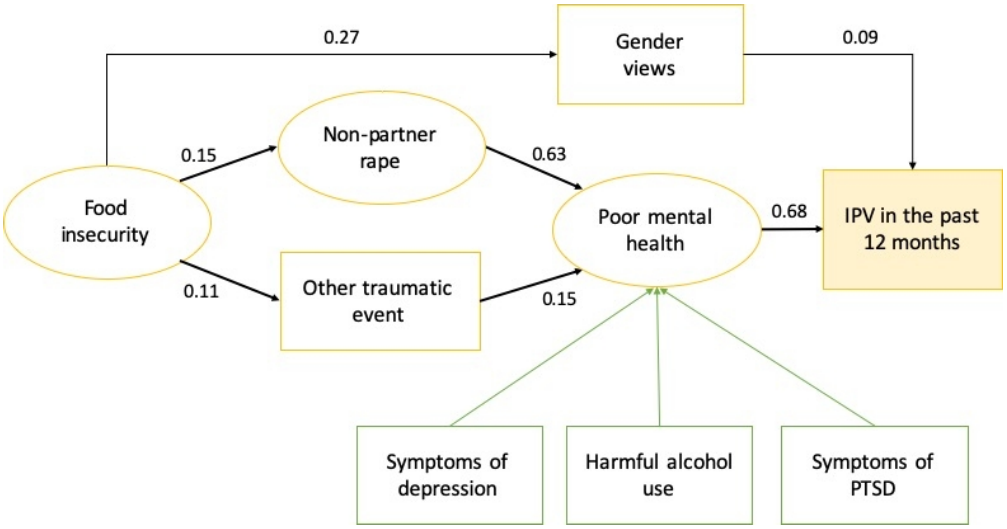

Structural equation model of pathways between exposure to traumatic events and IPV in the past 12 months (standardized coefficients)

277x144mm (72 x 72 DPI)

Reporting checklist for cross sectional study.

Based on the STROBE cross sectional guidelines.

Instructions to authors

Complete this checklist by entering the page numbers from your manuscript where readers will find each of the items listed below.

Your article may not currently address all the items on the checklist. Please modify your text to include the missing information. If you are certain that an item does not apply, please write "n/a" and provide a short explanation.

Upload your completed checklist as an extra file when you submit to a journal.

In your methods section, say that you used the STROBE cross sectionalreporting guidelines, and cite them as:

von Elm E, Altman DG, Egger M, Pocock SJ, Gotzsche PC, Vandenbroucke JP. The Strengthening the Reporting of Observational Studies in Epidemiology (STROBE) Statement: guidelines for reporting observational studies.

		Reporting Item	Page Number
**Title and abstract**			
Title	#1a	Indicate the study's design with a commonly used term in the title or the abstract	Page 1
Abstract	#1b	Provide in the abstract an informative and balanced summary of what was done and what was found	Page 2
**Introduction**			
Background / rationale	#2	Explain the scientific background and rationale for the investigation being reported	Page 5-6
Objectives	#3	State specific objectives, including any prespecified hypotheses	Page 6
**Methods**			
Study design	#4	Present key elements of study design early in the paper	Page 7

For peer review only

Setting	#5	Describe the setting, locations, and relevant dates, including periods of recruitment, exposure, follow-up, and data collection	Page 7
Eligibility criteria	#6a	Give the eligibility criteria, and the sources and methods of selection of participants.	Page 7
	#7	Clearly define all outcomes, exposures, predictors, potential confounders, and effect modifiers. Give diagnostic criteria, if applicable	Page 7
Data sources / measurement	#8	For each variable of interest give sources of data and details of methods of assessment (measurement). Describe comparability of assessment methods if there is more than one group. Give information separately for for exposed and unexposed groups if applicable.	7-9
Bias	#9	Describe any efforts to address potential sources of bias	7, 17
Study size	#10	Explain how the study size was arrived at	7
Quantitative variables	#11	Explain how quantitative variables were handled in the analyses. If applicable, describe which groupings were chosen, and why	7-9
Statistical methods	#12a	Describe all statistical methods, including those used to control for confounding	9-11
Statistical methods	#12b	Describe any methods used to examine subgroups and interactions	9-11
Statistical methods	#12c	Explain how missing data were addressed	NA
Statistical methods	#12d	If applicable, describe analytical methods taking account of sampling strategy	NA
Statistical methods	#12e	Describe any sensitivity analyses	9-11
**Results**			
Participants	#13a	Report numbers of individuals at each stage of study—eg numbers potentially eligible, examined for eligibility, confirmed eligible, included in the study, completing follow-up, and analysed. Give information separately for for exposed and unexposed groups if applicable.	NA

For peer review only

Participants	#13b	Give reasons for non-participation at each stage	NA
Participants	#13c	Consider use of a flow diagram	NA
Descriptive data	#14a	Give characteristics of study participants (eg demographic, clinical, social) and information on exposures and potential confounders. Give information separately for exposed and unexposed groups if applicable.	11-12
Descriptive data	#14b	Indicate number of participants with missing data for each variable of interest	NA
Outcome data	#15	Report numbers of outcome events or summary measures. Give information separately for exposed and unexposed groups if applicable.	11-13
Main results	#16a	Give unadjusted estimates and, if applicable, confounder-adjusted estimates and their precision (eg, 95% confidence interval). Make clear which confounders were adjusted for and why they were included	14
Main results	#16b	Report category boundaries when continuous variables were categorized	7-9
Main results	#16c	If relevant, consider translating estimates of relative risk into absolute risk for a meaningful time period	NA
Other analyses	#17	Report other analyses done—e.g., analyses of subgroups and interactions, and sensitivity analyses	14-15
**Discussion**			
Key results	#18	Summarise key results with reference to study objectives	15-17
Limitations	#19	Discuss limitations of the study, taking into account sources of potential bias or imprecision. Discuss both direction and magnitude of any potential bias.	17
Interpretation	#20	Give a cautious overall interpretation considering objectives, limitations, multiplicity of analyses, results from similar studies, and other relevant evidence.	15-18
Generalisability	#21	Discuss the generalisability (external validity) of the study results	17

For peer review only

**Other Information**			
Funding	#22	Give the source of funding and the role of the funders for the present study and, if applicable, for the original study on which the present article is based	19

None The STROBE checklist is distributed under the terms of the Creative Commons Attribution License CC-BY. This checklist can be completed online using https://www.goodreports.org/, a tool made by the EQUATOR Network in collaboration with Penelope.ai

For peer review only

BMJ Open

Does experiencing a traumatic life event increase the risk of intimate partner violence for young women? A cross sectional analysis and structural equation model of data from the Stepping Stones and Creating Futures intervention in South Africa

Journal:	*BMJ Open*
Manuscript ID	bmjopen-2021-051969.R1
Article Type:	Original research
Date Submitted by the Author:	31-Jan-2022
Complete List of Authors:	Mannell, Jenevieve ; UCL, Institute for Global Health<br>Minckas, Nicole; UCL, Institute for Global Health<br>Burgess, Rochelle; UCL, Institute for Global Health ; University of Johannesburg, Department of Social Work<br>Chirwa, Esnat; South African Medical Research Council, Gender and Health Division ; University of Witwatersrand, School of Public Health<br>Jewkes, Rachel; South African Medical Research Council, Gender and Health Division<br>Gibbs, Andrew; South African Medical Research Council, Gender and Health Division
**Primary Subject Heading**:	Global health
Secondary Subject Heading:	Mental health
Keywords:	Public health < INFECTIOUS DISEASES, Anxiety disorders < PSYCHIATRY, Substance misuse < PSYCHIATRY, Epidemiology < TROPICAL MEDICINE

SCHOLARONE™
Manuscripts

I, the Submitting Author has the right to grant and does grant on behalf of all authors of the Work (as defined in the below author licence), an exclusive licence and/or a non-exclusive licence for contributions from authors who are: i) UK Crown employees; ii) where BMJ has agreed a CC-BY licence shall apply, and/or iii) in accordance with the terms applicable for US Federal Government officers or employees acting as part of their official duties; on a worldwide, perpetual, irrevocable, royalty-free basis to BMJ Publishing Group Ltd ("BMJ") its licensees and where the relevant Journal is co-owned by BMJ to the co-owners of the Journal, to publish the Work in this journal and any other BMJ products and to exploit all rights, as set out in our licence.

The Submitting Author accepts and understands that any supply made under these terms is made by BMJ to the Submitting Author unless you are acting as an employee on behalf of your employer or a postgraduate student of an affiliated institution which is paying any applicable article publishing charge ("APC") for Open Access articles. Where the Submitting Author wishes to make the Work available on an Open Access basis (and intends to pay the relevant APC), the terms of reuse of such Open Access shall be governed by a Creative Commons licence – details of these licences and which Creative Commons licence will apply to this Work are set out in our licence referred to above.

Other than as permitted in any relevant BMJ Author's Self Archiving Policies, I confirm this Work has not been accepted for publication elsewhere, is not being considered for publication elsewhere and does not duplicate material already published. I confirm all authors consent to publication of this Work and authorise the granting of this licence.

For peer review only

Full article title:

Does experiencing a traumatic life event increase the risk of intimate partner violence for young women? A cross sectional analysis and structural equation model of data from the *Stepping Stones and Creating Futures* intervention in South Africa

Each author's complete name and institutional affiliation(s):

Jenevieve Mannell, Institute for Global Health, UCL, https://orcid.org/0000-0002-7456-3194

Nicole Minckas, Institute for Global Health, UCL, https://orcid.org/0000-0002-2650-443X

Rochelle A Burgess, Institute for Global Health, UCL, https://orcid.org/0000-0001-9749-7065

Esnat Chirwa, Gender and Health Research Unit, South African Medical Research Council, https://orcid.org/0000-0003-0471-4978

Rachel Jewkes, Gender and Health Research Unit, South African Medical Research Council, https://orcid.org/0000-0002-4330-6267

Andrew Gibbs, Gender and Health Research Unit, South African Medical Research Council, https://orcid.org/0000-0003-2812-5377

Corresponding author (name, address, phone/fax, e-mail): Jenevieve Mannell, Institute for Global Health, UCL, 30 Guilford Street, WC1N 1EH, j.mannell@ucl.ac.uk

For peer review only

Abstract

Objectives: To investigate associations and potential pathways between women's lifetime exposure to traumatic events and their recent experiences of intimate partner violence (IPV).

Setting: South African informal settlements near Durban.

Participants: 677 women, living in informal settlements, aged 18-30 years, currently out of school or formal employment.

Primary and secondary outcome measures: Self-reported experiences of IPV in the past 12 months and exposure to traumatic neighbourhood events (including witnessing murder, being robbed or kidnapped, witnessing and experiencing rape).

Results: Exposure to traumatic events was common among the 677 women surveyed. Over 70% had experienced at least one in their lifetime; one quarter (24%) had experienced 3 or more different events. Women exposed to any traumatic event had a 43% increase in the odds of experiencing IPV in comparison to those with no exposure (aOR1.43, P=<0.000). Exposure to non-partner rape is more strongly associated with IPV than any other traumatic experience. Pathways from exposure to traumatic events and non-partner rape to recent IPV experience are mediated by a latent variable of poor mental health. Food insecurity is associated with all forms of traumatic experience, and is also indirectly associated with IPV through views by women that are unsupportive of gender equality.

Conclusions: Women living in South African informal settlements who witness or experience traumatic events were likely to experience IPV, and this increases when women were exposed to multiple types of events. Our model suggests that experiencing traumatic events, and non-partner rape in particular, has negative effects on women's mental health in ways that may increase their vulnerability to IPV. IPV prevention interventions should consider the broader impacts of women's exposure to neighbourhood violence and severe poverty on IPV risk in settings where these are endemic.

For peer review only

Key words: intimate partner violence, non-partner rape, neighbourhood violence, trauma, mental health, South Africa

Strengths and Limitations of this study

- This study was the first, to our knowledge, to assess associations and potential pathways between young women's experiences of traumatic events (e.g. being robbed at gun point, witnessing murder) in neighbourhoods with a high incidence of crime and the odds that they will experience recent intimate partner violence;
- Structural equation modelling provides insight into how mental health indicators mediate the relationships between young women's exposure to traumatic events and intimate partner violence, which would not have been possible using logistic regression alone;
- Study participants were randomly sampled as part of baseline data collection for a cluster randomised controlled trial and is therefore representative of the young women living in South Africa's informal settlements;
- The study relies on self-reported measures of symptoms of post-traumatic stress disorder (PTSD) and depression rather than a formal diagnosis; the results therefore reflect women's understanding of symptoms of stress and anxiety and not PTSD or depression as defined in the *Diagnostic and Statistical Manual of Mental Disorders (DSM-5)*;
- From the questions women were asked about traumatic events in their lives, we were only able to assess whether women had experienced multiple types of events and not how many times each event may have been experienced by an individual.

For peer review only

Introduction

Experiencing or witnessing violence can have long-term traumatising effects that undermine women's mental health and wellbeing (1–3). Experiencing multiple types of violence, such as child abuse, intimate partner violence (IPV) and/or armed conflict, has been shown to compound these negative health effects (4–8). Our aim in this article is to contribute a better understanding of the cumulative effects of these different forms of violence and how these play out in the complex lives of women living in economically constrained settings (9,10). This responds to calls to explore the intersectional forms of violence at work in women's lives (11), including the simultaneous impacts of a range of structural violence(s), such as poverty, racism xenophobia and exclusion (12), and their negative mental health consequences (13).

Many women live in dangerous environments where the potential of experiencing community-based violence is extremely high, and studies suggest that this is associated with IPV experience. For instance, women living in settings either experiencing or that have recently experienced armed conflict are at a significantly higher risk of also experiencing IPV (7,14,15). Investigating IPV in Liberia, Kelly and colleagues find that women living in districts experiencing conflict were almost three times as likely to experience IPV compared to individuals in districts without conflict, even four years after peace was declared (16). Studies of women's experiences during armed conflict point to potential pathways through which an experience of war and conflict increases IPV. This includes poor mental health, which increases women's vulnerability to IPV as well as magnifying the negative outcomes of violence (17), and the role of stress and other symptoms of PTSD in increasing men's use of violence in the household (18–21). These risk factors for IPV are often linked theoretically to the gender inequalities that drive violence against women and girls, which are magnified by the highly stressful experiences of people affected by armed conflict (22,23).

For peer review only

Far less in known about the impact of violence in settings which are not conflict or post-conflict settings, but where exposure to traumatic events – such as being robbed at knifepoint, witnessing a murder or experiencing rape – are incredibly common, and the association of these experiences to IPV. Informal settlements in South Africa have a far higher incidence of traumatic events than in the rest of the country, and than many other countries around the world (24,25). In a study of men's exposure to traumatic life events in informal settlements in South Africa, witnessing or experiencing violent events was common with nearly a quarter of men having witnessed the murder of a family or friend (26). In these same informal settlements, two-thirds (65.2%) of women are estimated to have experienced IPV (27). The frequency with which people experience violence in informal settlements contributes to its normalisation, which is rooted in longstanding economic, racial and gender inequalities in South African society (28). This normalisation of violence in turn impacts mental health by upholding toxic masculinities that position strong men as desirable, and delegitimising the stress and fear women feel when experiencing acts of violence (29,30).

To further understand intersections between different forms of violence in women's lives, we investigated the association between women's lifetime exposure to different types of traumatic events and their recent experiences of IPV (past 12 months). We set out to test three main hypotheses: (1) women who experience more types of traumatic events have a higher risk of experiencing IPV, (2) there is a dose relationship between exposure to traumatic events and IPV (e.g. women who experience more types of traumatic events are more likely to experience IPV), (3) the relationship between exposure to traumatic events and IPV experiences is mediated by poor mental health.

Methods

Design

For peer review only

Our analysis draws on women's data collected as part of the baseline for Stepping Stones and Creating Futures – a cluster randomised controlled trial of an intervention to reduce IPV and HIV vulnerability among young people living in urban informal settlements in South Africa (31,32). Baseline data were collected between September 2015 and September 2016 in urban informal settlements near Durban (33).

Women included in the Stepping Stones and Creating Futures (SS/CF) trial were 18 to 30 years old, out of school and not currently in formal employment. They were also residents of the informal settlement, although the length of time they had lived in the settlement varied. All participants were recruited by Project Empower, a local NGO with over 20 years of experience working on participatory programming in South Africa's informal settlements. Ethical approval was obtained from the University of Kwa-Zulu Natal and the South African Medical Research Council. Women were recruited by Project Empower through staff walking around the community and talking to potential participants, as well as referral from other participants. The project was explained in detail to participants by trained data collectors, who were not Project Empower staff, and everyone was given time to ask questions about the research before giving their written consent to participate in the survey.

Data collection

The trial included 34 clusters with 19-21 women per cluster. All trial participants completed baseline questionnaires on a mobile phone in English, isiZulu or Xhosa, with the support of same-sex fieldworkers to assist in cases of low literacy. Questionnaires were tailored for women (a separate questionnaire was provided for men) and had logical skips patterns to facilitate survey completion. Questionnaires took between 45 minutes and 1.5 hours to complete. Participants were reimbursed for completing the questionnaire: R100 (~US$7 for those in the intervention arm and R300 (~US$21) in the control arm, because of the differential benefit of being involved in the intervention.

For peer review only

Patient and Public Involvement

The local partner organisation, Project Empower, participated in framing the research questions and planning the SS/CF intervention. They were active collaborators in the study, including quantitative and qualitative data collection and formulating recommendations from the analysis. Project Empower engaged community networks to ensure permission for community access and provided feedback to community leaders and organisations on the results of the study. The results of the trial have been disseminated to the communities involved. Our analysis will also be shared with young people living in informal settlements as part of a broader commitment to their active participation in interpreting findings about the health issues that affect their lives (34,35).

Measures

IPV was assessed using eight behaviourally specific questions about women's experiences, five about physical IPV and three about sexual IPV, with response options for each item: "never", "once" "few" or "many times". These questions were based on the WHO multi-country study survey on violence against women (36), which has been adapted for South Africa (37). A positive response to any of the questions was coded as having experienced IPV in the past 12 months.

Traumatic experiences, as the exposure variable, was compiled from twelve questions about women's past experiences of traumatic events in their lifetime. This included six questions about witnessing the murder of a friend/ family member, witnessing the murder of a stranger, witnessing an armed attack, being robbed at knife or gun point, being kidnapped, and witnessing someone being raped, and six questions about experiences of non-partner rape (forced sex, attempted forced sex, forced sex while under the influence of alcohol or drugs, forced sex by multiple men at the same time, forced sex by multiple men at the same time while under the influence of alcohol or drugs, and forced sex after agreeing to have sex with

For peer review only

someone else). Possible responses to each item were yes or no. We generated a binary variable for any non-partner rape that combined the six related items. We then summarised women's tramautic experiences in three ways. First, we generated a binary variable for never/ any experience of trauma. Second, we generated a variable classifying women into those who had (1) never experienced a traumatic event, (2) experienced 1-2 types of events, and (3) experienced 3 or more types of traumatic events. Third, we generated a variable that summed all the types of trauma into a score (range 0-7).

Women's mental health was assessed via measures of depressive symptoms, symptoms of post-traumatic stress disorder (PTSD), and alcohol use. For depressive symptoms, the Centre for Epidemiological Studies Depression Scale (CES-D) was used, which asks 20 questions about depressive symptoms in the past week with responses on a four-point Likert scale ranging from 'never' to 'most or all of the time' (38). Values associated with the Likert scale were summed to create a CES-D score (range 0–57, α=0.88). Symptoms of PTSD were assessed using the Harvard Trauma Questionnaire (39), which includes 16 questions about feelings in the past week related to traumatic events in the past with responses on a four-point Likert scale ranging from 'not at all' to 'extremely.' Values were summed to create a PTSD score (range 0–45, α=0.9), with a clinical cut-off score of 2.5. Harmful alcohol use was assessed using the Alcohol Use Disorder Identification Test (AUDIT) scale, which asks ten questions about past year alcohol use. Values associated with the Likert scale were summed to create an AUDIT score (range 0–38, α=0.81). Harmful alcohol use was defined as an AUDIT score of eight or more (40).

Other measures included demographic data on age, education level (primary/ secondary), and relationship status (married/ living with a partner, boyfriend, no relationship). Food insecurity was assessed using the Household Hunger Scale (41), which

For peer review only

asks how often in the past month there was no food to eat in their household, how often a member of the household had gone to sleep hungry, and how often a member of the household had not eaten for a whole day and night because of no food. Possible responses were on a four-point Likert scale (never, sometimes, often, very often), and summed values were recoded as three categorical variables: little or no hunger/ moderate hunger/ severe hunger. Gender views were assessed using the Gender Equitable Men's scale adapted for South Africa. This included 20 statements about the role and expectations of men and women with a four-point Likert scale from 'Strongly disagree' to 'Strongly agree.' Higher scores correspond to views that are less supportive of gender equality (range 2–54, α=0.86).

Analysis

All analyses were performed in STATA 16.1 and considered clustering of the survey data using the SVY command. We first provide descriptive statistics for the sample providing numbers and percentages. Second, we calculated the prevalence of the eight traumatic events asked about in the survey, and the various sums for overall trauma experiences.

We then carried out descriptive analyses of the relationship between socio-economic and mental health data and exposure to traumatic events. We provide percentages and 95% confidence intervals, or mean and 95%CI as appropriate and tested for significant differences using t-tests for continuous variables and chi-squared tests for categorical variables and report p-values.

We used logistic regression to estimate unadjusted and adjusted associations between exposure to traumatic events and IPV experiences in the past 12 months. We first assessed whether each individual traumatic event was associated with IPV, then whether never/any, then never, 1, 2, 3+. We reported odds ratios, 95%CI and p-values. Adjusted models included the covariates age, education, relationship status, harmful alcohol use, food insecurity and gender views. Due to odds ratios over-inflating the association between variables with non-

For peer review only

rare events, we re-ran the analysis as a supplementary analysis, using poisson regression to calculate relative risk ratios, using the same modelling approach as for logistic regression.

Second, we built a structural equation model (SEM) to assess whether there were direct and mediated pathways between lifetime traumatic events and past 12 month IPV experience. To build the SEM, we first identified potential pathways drawing on the theoretical literature available. Given the qualitative difference between being raped oneself and witnessing a traumatic event (such as murder or rape), we constructed a latent variable for non-partner rape and separated this from other experiences of traumatic events. We then regressed each variable included in the draft model to all other variables they were hypothesised to be associated with (i.e. continuous outcome scores for food insecurity, gender views, depression, PTSD, and harmful alcohol use), and included only those pathways that were significant ($P<0.05$). Based on a previous analysis of the same dataset (27), we constructed a latent variable for food insecurity by separating questions from the household hunger scale into three distinct variables as part of the model. We then assessed how well our model predicted the sample variance-covariance matrix by examining indices of model fit including the standardized root mean squared residual (SRMR <0.1), root mean squared error of approximation (RMSEA <0.05), comparative fit index (CFI >0.95), and Tucker-Lewis Index (TLI >0.95). We modified the model using the MINDICES command in STATA to identify correlations between error terms with a good trade-off between improved model fit and an increase in degrees of freedom (e.g. modification indices >3.84). We then covaried error terms where we could justify this theoretically.

Results

At baseline, 677 young women were recruited and provided data. Roughly one third of the women (30.4%) had completed secondary education (Table 1). Two-thirds were in a relationship: 63.5% had a boyfriend but were not living with them, and 18.2% were either

For peer review only

living with a partner or married. Food insecurity was also common with 50.1% of women living in households experiencing moderate food insecurity, and an additional 31.3% with severe food insecurity. Approximately one fifth of women experienced poor mental health: 21% of women were above the mean of 2.5 on the PTSD scale; 45.2% met the cut-off measure of 20/21 for depression; and 23% of women met the cut-off measure of eight or more for harmful alcohol use.

Table 1: Women's socio-demographic and baseline characteristics, by exposure to traumatic events

	n	*%*	No traumatic experience (n=185) *Mean/% [95% CI]*	1-2 traumatic experiences (n=326) *Mean/% [95% CI]*	3+ traumatic experiences (n=166) *Mean/% [95% CI]*	P-value
**Age**						0.97
18-20	142	21.0%	20.0 [14.8, 26.4]	22.09 [17.9, 26.9]	19.9 [14.5, 26.7]	
21-25	313	46.2%	47.6 [40.4, 54.8]	45.4 [40.0, 50.9]	46.4 [38.9, 54.1]	
26-30	222	32.8%	32.4 [26.0, 39.6]	32.5 [27.6, 37.8]	33.73 [26.9, 41.3]	
**Education**						0.02
Secondary complete	206	30.4%	62.7 [55.7, 69.3]	69.9 [64.8, 74.6]	76.5 [69.5, 82.3]	
Secondary incomplete/ no secondary	471	69.6%	37.3 [30.8, 44.3]	30.1 [25.4, 35.2]	23.5 [17.7, 30.5]	
**Relationship Status**						0.82
Married/ Cohabitating	123	18.2%	18.9 [13.9, 25.2]	17.8 [14.0, 22.3]	18.1 [12.9, 24.7]	
Boyfriend	430	63.5%	62.7 [55.5, 69.4]	65.3 [60.0. 70.3]	60.8 [53.1, 68.0]	
No relationship	124	18.3%	18.4 [13.5, 24.5]	16.87 [13.2, 21.3]	21.1 [15.5, 28.0]	
**Food insecurity**						0.16
Mild (no/little hunger)	126	18.6%	22.7 [17.3, 29.2]	17.8 [14.0, 22.3]	15.7 [10.9, 22.0]	
Moderate	339	50.1%	51.9 [44.6, 59.1]	50.3 [44.9, 55.7]	47.6 [40.1, 55.2]	
Severe	212	31.3%	25.4 [19.6, 32.2]	31.9 [27.0, 37.2]	36.8 [29.8, 44.3]	
**Common mental disorders**						
Depression (score)*	677	NA	17.4 [16.0, 18.8]	22.3 [21.1, 23.4]	23.1 [21.4, 24.8]	
PTSD (score)*	677	NA	1.6 [1.5, 1.7]	1.9 [1.9, 2.0]	2.1 [2.0, 2.2]	
Alcohol abuse (score)*	677	NA	0.1 [0.1, 0.2]	0.2 [0.2, 0.3]	0.3 [0.3, 0.4]	
**Gender attitudes**						
(score)	677	NA	25.2 [23.8, 26.5]	26.0 [25.0, 26.0]	25.4 [23.9, 27.0]	

*p-value <0.001: Levels of significance for continuous variables calculated using t-tests (no trauma experience as comparison)

For peer review only

Lifetime experience of traumatic events was common in the lives of these women (Table 2). Over a third had been robbed at knife or gun point (35.2%), and roughly the same number had witnessed an armed attack (34.6%). A quarter (26.1%) had witnessed the murder of a stranger or someone they know, while almost a fifth (17.6%) had witnessed the murder of a friend or family member. One in ten (10.5%) had witnessed someone being raped, and nearly a third (30.4%) had been raped themselves. Over 70% of women had experienced at least one of these traumatic events, while 24% had experienced three or more different types.

Table 2: Proportion of women who experienced traumatic events by type of event and frequency

	N	% (95% CI)
**By type of traumatic event**		
Witnessed murder friend	119	17.6 [14.7, 20.5]
Witnessed murder stranger	117	26.1 [22.8, 29.5]
Witnessed armed attack	234	34.6 [31.0, 38.2]
Robbed at gun/ knife point	238	35.2 [31.5, 38.8]
Kidnapped	32	4.7 [3.1, 6.3]
Witnessed rape	71	10.5 [8.2, 12.8]
Experienced non-partner rape	205	30.3 [26.8, 33.8]
**Multiple traumatic events**		
Yes	492	72.7 [69.3, 76.0]
**Number of different traumatic events**		
None	185	27.3 [24.1, 30.8]
1 traumatic event	209	30.87 [27.5, 34.5]
2 traumatic events	117	17.28 [14.6, 20.3]
3 or more traumatic events	166	24.5 [21.4, 27.9]

While there is a significant difference in the number of different traumatic events experienced by women who have completed secondary education in comparison to those who have not, there is not a significant difference according to age, relationship status or food insecurity. Gender views are also not significantly associated with an increase in exposure to different traumatic events. However, the variables signifying common mental disorders (depression, symptoms of PTSD, and harmful alcohol use) are all significantly associated with an increase in the number of different traumatic experiences.

Table 3 reports results from the logistic regression. Non-partner rape is the only traumatic experience that has a significant association with IPV experience in the past year when controlling for age, education, relationship status, food insecurity, harmful alcohol use

For peer review only

and gender views. Women exposed to any traumatic event other than experiencing non-partner rape have a 43% increase in the odds of experiencing IPV in the past year in the adjusted analysis, compared to those with no trauma exposure (aOR 1.43). When non-partner rape is included as a traumatic experience in the model, women have an 83% increase in the odds of experience IPV in the past year, compared to those with no trauma exposure (aOR 1.83). There also appears to be a relationship whereby the odds of experiencing IPV increases as women are exposed to one or two types of traumatic events in comparison to those who did not experience any such events (1 event, aOR 1.66; 2 events, aOR 2.47). However, this dose relationship between exposure to different traumatic events and IPV experience tapers off when more than two types of traumatic events have been experienced (3+ events, aOR 1.71). The Poisson regression shows the same associations in unadjusted and adjusted models, with the relative risk ratios being lower in magnitude than the logistic regression.

Table 3: Associations between exposure to traumatic events and IPV in the past 12 months

Variable	Logistic		Poisson	
	**OR (95% CI)**	**aOR (95% CI)**	**RRR(95%CI)**	**aRRR(95%CI)**
**Individual traumatic event**				
Witnessed murder friend	1.23 (0.80,1.88)	1.00 (0.63,1.59)	1.07 (0.93,1.23)	1.02 (0.89,1.16)
Witnessed murder stranger	1.02 (0.71,1.47)	0.92 (0.62,1.37)	1.01 (0.89,1.14)	0.98 (0.87,1.11)
Witnessed armed attack	**1.40 (1.00,1.97)***	1.34 (0.93,1.92)	**1.12 (1.00,1.25)***	1.10 (0.99,1.23)
Robbed at gun/ knife point	1.37 (0.98,1.92)	1.19 (0.82,1.76)	1.11 (1.00,1.24)	1.06 (0.95,1.19)
Kidnapped	1.64 (0.72,3.73)	0.85 (0.36,1.93)	1.16 (0.94,1.43)	0.97 (0.79,1.18)
Witnessed rape	**1.96 (1.09,3.51)***	1.58 (0.85,2.92)	**1.22 (1.06,1.40)****	1.13 (0.98,1.29)
Experienced non-partner rape	**2.82 (1.91,4.16)*****	**2.35 (1.54,3.57)*****	**1.36 (1.23,1.51) *****	**1.27 (1.14,1.41) *****
**Multiple traumatic events**				
Any trauma (excluding non-partner rape)	**1.61 (1.16,2.23)****	**1.43 (1.00,2.04)***	**1.19 (1.05,1.35)****	**1.14 (1.01,1.28)***
Any trauma (including non-partner rape)	**2.11 (1.49,2.98)*****	**1.83 (1.26,2.66)****	**1.33 (1.15,1.55) *****	**1.25 (1.08,1.45)****
**Number of different traumatic events**				
1 traumatic event	**1.76 (1.17,2.65)*****	**1.66 (1.08,2.57)***	**1.26 (1.06,1.49) ****	**1.22 (1.03,1.43) ***
2 types of traumatic events	**2.88 (1.74,4.78)*****	**2.47 (1.43,4.27)****	**1.45 (1.23,1.72) *****	**1.35 (1.14,1.60) ****
3+ types of traumatic events	**2.17 (1.39,3.36)*****	**1.71 (1.05,2.78)***	**1.34 (1.14,1.59) *****	**1.24 (1.05,1.46) ***

Model adjusted for age, education, relationship status, food insecurity, harmful alcohol use and gender views.

Note: Boldface indicates statistical significance (*p<0.05; **p<0.01; ***p<0.001).

For peer review only

Table 4 presents the direct associations between all variables in preparation for the SEM with significant associations (P<0.05) highlighted in bold. Nearly all associations were significant with the exception of the association between food insecurity and alcohol abuse, and several associations with gender attitudes (including exposure to traumatic events, alcohol abuse, depression and PTSD). These associations were removed from our SEM.

Table 4: Assessing potential pathways of association

	Traumatic events	IPV past 12 months	Non-partner rape	Food insecurity	Alcohol abuse	Depress	PTSD
IPV past 12 mo	**0.052**						
Non-partner rape	**0.687**	**0.028**					
Food insecurity	**0.052**	**0.091**	**0.020**				
Alcohol abuse	**0.040**	**0.356**	**0.021**	0.015			
Depression	**0.017**	**0.185**	**0.010**	**0.053**	**0.163**		
PTSD	**0.035**	**0.211**	**0.011**	**0.454**	**0.203**	**0.779**	
Gender views	0.003	**0.081**	0.002	**0.068**	0.019	0.023	0.039

Associations significant at 95%CI in **bold**

In the SEM, the importance of non-partner rape as a traumatic experience meant that it needed to be separated as a variable from other types of traumatic events to attain a good model fit. As shown in Figure 1, the association between exposure to traumatic events (including the covaried effects of non-partner rape) and IPV in the past 12 months was mediated by indicators of poor mental health, specifically symptoms of depression, PTSD and alcohol abuse, which were all increased by trauma experience and which were directly associated with increased IPV. Increased symptoms of poor mental health were also mediated by increased food insecurity. Food insecurity was also associated with IPV experience mediated by inequitable gender views. The SEM was a good fit with the data (RMSEA 0.046; CFI 0.97; TLI 0.961). All standardised coefficients for the SEM are presented in Table 5.

Table 5: Structural equation model

Parameter Direct effects	Standardized coefficients	SE	z	P>|z|	95% confidence interval	
Food insecurity→Trauma	0.11	0.04	2.70	0.007	0.03	0.19
Food insecurity→ Non-partner rape	0.15	0.04	3.58	0.000	0.07	0.24
Food insecurity→Gender views	0.27	0.04	7.01	0.000	0.19	0.34
Trauma→Poor mental health	0.15	0.04	3.36	0.000	0.06	0.23
Non-partner rape→Poor mental health	0.63	0.04	15.43	0.000	0.55	0.71
Poor mental health→IPV	0.68	0.03	20.65	0.000	0.62	0.75
Gender views→IPV	0.09	0.03	2.69	0.007	0.02	0.15
**Latent variable: Non-partner rape**						
Forced sex	0.65	0.03	25.28	0.000	0.59	0.70
Attempted forced sex	0.54	0.03	17.58	0.000	0.48	0.59
Forced sex, alcohol or drugs	0.75	0.02	32.42	0.000	0.71	0.79
Forced sex, multiple men	0.71	0.02	30.30	0.000	0.67	0.76
Forced sex, multiple men, alcohol or drugs	0.82	0.08	45.00	0.000	0.79	0.86
Forced sex after agreeing with someone else	0.69	0.02	27.92	0.000	0.64	0.74
**Latent variable: Poor mental health**						
Symptoms of depression	0.45	0.04	11.63	0.000	0.38	0.53
Harmful alcohol use	0.61	0.03	17.51	0.000	0.54	0.67
Symptoms of PTSD	0.49	0.04	13.27	0.000	0.43	0.57
**Latent variable: Food insecurity**						
No food to eat	0.78	0.02	32.70	0.000	0.68	0.77
Going to sleep hungry	0.93	0.02	54.15	0.000	0.89	0.96
24hrs without eating	0.75	0.02	35.14	0.000	0.71	0.79

Figure 1: Structural equation model of pathways between exposure to traumatic events and IPV in the past 12 months (standardized coefficients)

Discussion

These results support our hypothesis that women living in informal settlements who experience traumatic events are at a higher risk of experiencing IPV. There was also a large effect size for those experiencing two or more different traumatic events, in comparison to only one event. This acknowledges how women's exposure to multiple and overlapping violence(s) (including violence outside of the household) impacts their risk of IPV and poor mental health outcomes for women, building on the already well-recognised consideration of the role of men's exposure to violence in their perpetration of IPV (14,42).

For peer review only

Both the logistic regression and the SEM highlight the overwhelming importance of non-partner rape as a traumatic event in women's risk of experiencing IPV. Non-partner rape is more strongly associated with IPV experience than any other traumatic event assessed in our analysis. This highlights the importance of non-partner rape in women's risk of experiencing IPV, confirming findings from other studies that have highlighted associations between non-partner rape and an increased risk of IPV (43). This may be explained by the devastating consequences of non-partner rape for women's mental health and the importance of self-blame (44) and disengagement coping strategies (i.e. passive reactions and avoidance (45) in increasing their vulnerability to other forms of violence (46). It also points to a need for further research to investigate positive mental health supports (i.e. engagement coping strategies, gender awareness, etc) that could be provided to women following experiences of non-partner rape that may help in reducing their risk of IPV. For example, recent work has highlighted the ability for such interventions to reduce symptoms of poor mental health, as well as increase self-efficacy and collective agency, in response to the social-structural challenges that increase women's risk of IPV (including food insecurity) (47).

Our analysis also shows the importance of indicators of poor mental health (including depression, symptoms of PTSD, and harmful alcohol use) in mediating the relationship between experiencing traumatic events and IPV, as shown in the SEM. This is consistent with literature on the role of symptoms of PTSD in increasing women's risk of physical and sexual revictimization (46,48), and confirms findings from other studies of the importance of poor mental health and substance abuse as risk factors for IPV (49,50). While our analysis has focused on poor mental health resulting from exposure to interpersonal neighbourhood-based acts of violence, it supports findings from other studies that have examined the increased IPV risk of exposure to traumatic events such as natural disasters (51,52). The evidence on interpersonal violence and disasters are similar in suggesting that the relationship

For peer review only - http://bmjopen.bmj.com/site/about/guidelines.xhtml

between poor mental health and IPV is bidirectional, whereby symptoms of poor mental health leads to IPV, which in turn worsens women's mental health (53,54).

Furthermore, our analysis shows how food insecurity (as a proxy for poverty) is a contributing factor in women's exposure to traumatic events. In the SEM, food insecurity plays a role in increasing both non-partner rape and other traumatic events in women's lives. Moreover, the association between food insecurity and IPV experience is mediated by gender views unsupportive of women's equality, a pathway corroborated by evidence that food insecurity may be an indicator of women's economic dependence on men with implications for their ability to leave violent relationships (27). The role of food insecurity as a compounding factor in women's exposure to trauma has important implications for thinking about poverty as an additional form of structural violence in women's lives (55), and how this in turn compounds the risk of IPV in women's relationships. This contributes important insights to current understandings of the effects of multiple experiences of trauma or *polyvictimisation* (56–58) by highlighting the need to explore violence happening at a structural as well as an interpersonal level.

Limitations

This study has some limitations. Our data relies on self-reported measures of traumatic events, mental health and IPV. In the case of IPV and traumatic events, questions have been framed around specific acts of violence (hitting, slapping, kicking, etc.) consistent with recognised best practice in the field of violence against women. However, this resulted in a limited number of questions being asked about the types of traumatic events women had experienced and likely did not reflect the full range of events that they potentially experience in their lives. The way in which questions about trauma were asked (e.g. *have you experienced any of this list of possible events?*) means that we were only able to assess whether women had experienced multiple types of events rather than how many times each

For peer review only

event may have been experienced by an individual. Moreover, the data does not tell us if the events women experienced were actually traumatising or if they led to a mental health disorder. The survey did not ask about diagnosis and self-reporting mental health measures are limited in their diagnostic potential. The study was also cross-sectional and women were not asked about when the traumatic event occurred in their life. It is therefore possible (although unlikely) that women's experiences of traumatic events happened more recently than their experience of IPV in the past year.

Conclusions

Our results highlight the need for interventions that comprehensively address multiple forms of violence, with particular attention to non-partner rape, as a means of reducing this traumatic event in and of itself, and as a means of reducing IPV. In settings with extremely high rates of violence, such as South Africa's informal settlements, women are often exposed to a range of traumatic events, and this has negative consequences for their mental health in ways that put them at increased risk of IPV. As a consequence, IPV interventions in these settings require comprehensive mental health strategies and supports as an integral part of intervention activities. Working through women's trauma is not only an optional add-on to IPV prevention interventions, but an integral resource for reducing women's risk of IPV. Attention to men's trauma is also important for IPV reduction interventions, particularly given the evidence that the men who commit non-partner rape are often the same as those who perpetuate IPV, and that this is often associated with their own experiences of child abuse and neglect (59). However, it is important to remember that attention to poor mental health as an IPV prevention strategy also needs to be considered alongside the urgent need for structural interventions that address the contexts of violence and poverty as a key driver of a multiple intersecting forms of violence in this context.

For peer review only

Contributorship Statement

JM and AG conceptualised the paper. The analysis was completed by JM with assistance from EC and AG, who verified the results at multiple time points. AG, RJ and EC were involved in the cluster RCT that provided the baseline data for this analysis. There was substantial intellectual input from RJ , NM and RB during multiple rounds of analysis and drafts of the article. RB also contributed specifically to the interpretation of the analysis of the mental health results.

Competing interests

None declared.

Funding

The initial study was funded through the What Works To Prevent Violence? A Global Programme on Violence Against Women and Girls (VAWG) funded by the UK Government's Department for International Development (DFID), and managed by the South African Medical Research Council. However, the views expressed do not necessarily reflect the department's official policies and the funders had no role in study design; collection, management, analysis, and interpretation of data; writing of the report; and the decision to submit the paper for publication. The secondary data analysis performed for this article was supported by UKRI MRC Global Challenge Research Fund MR/T029803/1 and UKRI Future Leaders Fellowship MR/S033629/1.

Data sharing

For peer review only

De-identified data set for this project is available from https://medat.samrc.ac.za/index.php/catalog/23 managed by the South African Medical Research Council.

Ethical approval

Ethical approval was obtained from the University of Kwa-Zulu Natal (BFC043/15) and the South African Medical Research Council (EC006-2/2015). All participants gave their informed consent to participate in the survey before taking part.

References

1. Caetano R, Cunradi C. Intimate partner violence and depression among Whites, Blacks, and Hispanics. Annals of epidemiology. 2003 Nov;13(10):661–5.

2. Erolin KS, Wieling E, Parra RE. Family violence exposure and associated risk factors for child PTSD in a Mexican sample. Child abuse & neglect. 2014 Jun;38(6):1011–22.

3. Shen AC. Long-term effects of interparental violence and child physical maltreatment experiences on PTSD and behavior problems: a national survey of Taiwanese college students. Child abuse & neglect. 2009 Mar;33(3):148–60.

4. Afifi TO, Taillieu T, Zamorski MA, Turner S, Cheung K, Sareen J. Association of child abuse exposure with suicidal ideation, suicide plans, and suicide attempts in military personnel and the general population in Canada. JAMA Psychiatry. 2016;73(3):229–38.

5. Chiu GR, Lutfey KE, Litman HJ, Link CL, Hall SA, McKinlay JB. Prevalence and overlap of childhood and adult physical, sexual, and emotional abuse: a descriptive analysis of results from the Boston Area Community Health (BACH) survey. Violence and victims. 2013;28(3):381–402.

For peer review only

6. de Jong JT, Komproe IH, Van Ommeren M, El Masri M, Araya M, Khaled N, et al. Lifetime events and posttraumatic stress disorder in 4 postconflict settings. Jama. 2001 Aug 1;286(5):555–62.

7. Gupta J, Falb KL, Carliner H, Hossain M, Kpebo D, Annan J. Associations between Exposure to Intimate Partner Violence, Armed Conflict, and Probable PTSD among Women in Rural Côte d'Ivoire. PLoS One [Internet]. 2014 May 13 [cited 2020 Mar 13];9(5). Available from: https://www.ncbi.nlm.nih.gov/pmc/articles/PMC4019518/

8. Kinyanda E, Weiss HA, Mungherera M, Onyango-Mangen P, Ngabirano E, Kajungu R, et al. Intimate partner violence as seen in post-conflict eastern Uganda: Prevalence, risk factors and mental health consequences Health and human rights of marginalized populations. BMC International Health and Human Rights [Internet]. 2016;16(1). Available from: https://www.scopus.com/inward/record.uri?eid=2-s2.0-84957109647&partnerID=40&md5=baae54ae31d02346e52cb6c08a50a16e

9. Guedes A, Bott S, Garcia-Moreno C, Colombini M. Bridging the gaps: a global review of intersections of violence against women and violence against children. Global health action. 2016;9:31516.

10. Heise L, Fulu E. State of the field of violence against women and girls: what do we know and what are the knowledge gaps. [Internet]. Pretoria: South African Medical Research Council.; 2014. Available from: https://assets.publishing.service.gov.uk/media/57a089b640f0b6497400021a/What_Works_Inception_Report_June_2014_AnnexD_WG1_paper_State_of_the_field.pdf

11. Kazdin AE. Conceptualizing the challenge of reducing interpersonal violence. Psychology of Violence. 2011;1(3):166–87.

For peer review only

12. Galtung J, Fischer D. Violence: Direct, Structural and Cultural. In: Galtung J, Fischer D, editors. Johan Galtung: Pioneer of Peace Research [Internet]. Berlin, Heidelberg: Springer; 2013 [cited 2021 Mar 15]. p. 35–40. (SpringerBriefs on Pioneers in Science and Practice). Available from: https://doi.org/10.1007/978-3-642-32481-9_3

13. Burgess RA, Fonseca L. Re-thinking recovery in post-conflict settings: Supporting the mental well-being of communities in Colombia. Global Public Health. 2020 Feb 1;15(2):200–19.

14. Annan J, Brier M. The risk of return: Intimate partner violence in Northern Uganda's armed conflict. Social Science & Medicine. 2010 Jan 1;70(1):152–9.

15. Jewkes R, Jama-Shai N, Sikweyiya Y. Enduring impact of conflict on mental health and gender-based violence perpetration in Bougainville, Papua New Guinea: A cross-sectional study. PLOS ONE. 2017 Oct 25;12(10):e0186062.

16. Kelly JTD, Colantuoni E, Robinson C, Decker MR. From the battlefield to the bedroom: a multilevel analysis of the links between political conflict and intimate partner violence in Liberia. BMJ Global Health. 2018 Apr 1;3(2):e000668.

17. Jewkes R, Corboz J, Gibbs A. Trauma exposure and IPV experienced by Afghan women: Analysis of the baseline of a randomised controlled trial. PLOS ONE. 2018 Oct 10;13(10):e0201974.

18. Colson E. War and domestic violence. Cultural survival quarterly. 1995 Spring;35–8.

19. Gibbs A, Said N, Corboz J, Jewkes R. Factors associated with 'honour killing' in Afghanistan and the occupied Palestinian Territories: Two cross-sectional studies. PLoS

For peer review only

One [Internet]. 2019 Aug 8 [cited 2020 Mar 10];14(8). Available from: https://www.ncbi.nlm.nih.gov/pmc/articles/PMC6687286/

20. Mannell J, Ahmad L, Ahmad A. Narrative storytelling as mental health support for women experiencing gender-based violence in Afghanistan. Social Science & Medicine. 2018 Oct 1;214:91–8.

21. Stark L, Roberts L, Wheaton W, Acham A, Boothby N, Ager A. Measuring violence against women amidst war and displacement in northern Uganda using the "neighbourhood method." Journal of Epidemiology and Community Health. 2010;64(12):1056–61.

22. Devakumar D, Palfreyman A, Uthayakumar-Cumarasamy A, Ullah N, Ranasinghe C, Minckas N, et al. Mental health of women and children experiencing family violence in conflict settings: a mixed methods systematic review. Conflict and Health. 2021 Oct 15;15(1):74.

23. Mannell J, Grewal G, Ahmad L, Ahmad A. A Qualitative Study of Women's Lived Experiences of Conflict and Domestic Violence in Afghanistan. Violence Against Women. 2020 Jul 6;1077801220935191.

24. Kaminer D, Plessis B du, Hardy A, Benjamin A. Exposure to Violence Across Multiple Sites Among Young South African Adolescents. Peace and Conflict: Journal of Peace Psychology. 2013 May;19(2):112–24.

25. Wakefield L, Tait S. Crime and Violence in Formal and Informal Urban Spaces in South Africa Citizen Security Dialogues Dispatches from South Africa: Foreword. Stability: International Journal of Security and Development. 2015 May 5;4(1):Art. 26.

For peer review only

26. Gibbs A, Hatcher A, Jewkes R, Sikweyiya Y, Washington L, Dunkle K, et al. Associations Between Lifetime Traumatic Experiences and HIV-Risk Behaviors Among Young Men Living in Informal Settlements in South Africa: A Cross-Sectional Analysis and Structural Equation Model. J Acquir Immune Defic Syndr. 2019 Jun 1;81(2):193–201.

27. Gibbs A, Jewkes R, Willan S, Washington L. Associations between poverty, mental health and substance use, gender power, and intimate partner violence amongst young (18-30) women and men in urban informal settlements in South Africa: A cross-sectional study and structural equation model. PLOS ONE. 2018 Oct 3;13(10):e0204956.

28. Lamb S, Snodgrass L. Growing up with normalised violence : narratives of South African youth. Commonwealth Youth and Development. 2013 Jan 1;11(1):4–21.

29. Gordon SF, Collins A. "We face rape. We face all things": Understandings of gender-based violence amongst female students at a South African university. African Safety Promotion: A Journal of Injury and Violence Prevention. 2013;11(2):93–106.

30. Willan S, Ntini N, Gibbs A, Jewkes R. Exploring young women's constructions of love and strategies to navigate violent relationships in South African informal settlements. Culture, Health & Sexuality. 2019 Jan 11;0(0):1–15.

31. Gibbs A, Washington L, Willan S, Ntini N, Khumalo T, Mbatha N, et al. The Stepping Stones and Creating Futures intervention to prevent intimate partner violence and HIV-risk behaviours in Durban, South Africa: study protocol for a cluster randomized control trial, and baseline characteristics. BMC Public Health. 2017 Apr 20;17(1):336.

For peer review only

[dataset] 32. Gibbs A, Chirwa E, Abdelatif N, Jewkes R. Stepping Stones and Creating Futures intervention trial data. 31 Jan 2022. Available from: http://medat.samrc.ac.za/index.php/catalog/WW

33. Gibbs A, Washington L, Abdelatif N, Chirwa E, Willan S, Shai N, et al. Stepping Stones and Creating Futures Intervention to Prevent Intimate Partner Violence Among Young People: Cluster Randomized Controlled Trial. Journal of Adolescent Health. 2020 Mar 1;66(3):323–35.

34. Mannell J, Willan S, Shahmanesh M, Seeley J, Sherr L, Gibbs A. Why interventions to prevent intimate partner violence and HIV have failed young women in southern Africa. Journal of the International AIDS Society. 2019;22(8):e25380.

35. Mannell J, Davis K. Evaluating Complex Health Interventions With Randomized Controlled Trials: How Do We Improve the Use of Qualitative Methods?: Qualitative Health Research [Internet]. 2019 Mar 14 [cited 2019 Mar 15]; Available from: https://journals.sagepub.com/eprint/jEbAMi4r9usX24a8mU4K/full

36. García-Moreno C, Jansen HAFM, Ellsberg M, Heise L, Watts C. WHO multi-country study on women's health and domestic violence against women: initial results on prevalence, health outcomes and women's responses. [Internet]. Geneva: World Health Organisation; 2005. Available from: https://www.who.int/reproductivehealth/publications/violence/24159358X/en/

37. Jewkes R, Nduna M, Levin J, Jama N, Dunkle K, Khuzwayo N, et al. A cluster randomized-controlled trial to determine the effectiveness of Stepping Stones in preventing HIV infections and promoting safer sexual behaviour amongst youth in the

For peer review only

rural Eastern Cape, South Africa: trial design, methods and baseline findings. Tropical Medicine & International Health. 2006;11(1):3–16.

38. Radloff LS. The CES-D Scale: A Self-Report Depression Scale for Research in the General Population. Applied Psychological Measurement. 1977 Jun 1;1(3):385–401.

39. Mollica RF, Caspi-Yavin Y, Bollini P, Truong T, Tor S, Lavelle J. The Harvard Trauma Questionnaire. Validating a cross-cultural instrument for measuring torture, trauma, and posttraumatic stress disorder in Indochinese refugees. Journal of nervous and mental disease. 1992;180(2):111–6.

40. Saunders JB, Aasland OG, Babor TF, Fuente JRDL, Grant M. Development of the Alcohol Use Disorders Identification Test (AUDIT): WHO Collaborative Project on Early Detection of Persons with Harmful Alcohol Consumption-II. Addiction. 1993;88(6):791–804.

41. Deitchler M, Ballard T, Swindale A, Coates J. Validation of a measure of household hunger for cross-cultural use. In Washington, D.C.: Academy for Educational Development; 2010.

42. Rees S, Thorpe R, Tol W, Fonseca M, Silove D. Testing a cycle of family violence model in conflict-affected, low-income countries: A qualitative study from Timor-Leste. Social Science and Medicine. 2015;130:284–91.

43. Jewkes R, Fulu E, Roselli T, Garcia-Moreno C. Prevalence of and factors associated with non-partner rape perpetration: findings from the UN Multi-country Cross-sectional Study on Men and Violence in Asia and the Pacific. The Lancet Global Health. 2013 Oct 1;1(4):e208–18.

For peer review only

44. Campbell R, Dworkin E, Cabral G. An Ecological Model of the Impact of Sexual Assault On Women's Mental Health. Trauma, Violence, & Abuse. 2009 Jul 1;10(3):225–46.

45. Dijkstra MTM, Homan AC. Engaging in Rather than Disengaging from Stress: Effective Coping and Perceived Control. Front Psychol. 2016 Sep 21;7:1415.

46. Iverson KM, Litwack SD, Pineles SL, Suvak MK, Vaughn RA, Resick PA. Predictors of Intimate Partner Violence Revictimization: The Relative Impact of Distinct PTSD Symptoms, Dissociation, and Coping Strategies. Journal of Traumatic Stress. 2013;26(1):102–10.

47. Burgess RA, Jeske N, Rasool S, Ahmad A, Kydd A, Ncube Mlilo N. Exploring the impact of a complex intervention for women with depression in contexts of adversity: A pilot feasibility study of COURRAGE-plus in South Africa. Int J Soc Psychiatry. 2021 Apr 15;207640211010203.

48. Sandberg DA, Matorin AI, Lynn SJ. Dissociation, Posttraumatic Symptomatology, and Sexual Revictimization: A Prospective Examination of Mediator and Moderator Effects. J Trauma Stress. 1999 Jan 1;12(1):127–38.

49. Avanci J, Assis S, Oliveira R. A cross-sectional analysis of women's mental health problems: Examining the association with different types of violence among a sample of Brazilian mothers. BMC Women's Health [Internet]. 2013;13(1). Available from: https://www.scopus.com/inward/record.uri?eid=2-s2.0-84876111818&partnerID=40&md5=1280a3617f5f2bb2b675f852c77b2392

50. Gibbs A, Dunkle K, Ramsoomar L, Willan S, Shai NJ, Chatterji S, et al. New learnings on drivers of men's physical and/or sexual violence against their female partners, and

For peer review only

women's experiences of this, and the implications for prevention interventions. Global Health Action. 2020 Dec 31;13(1):1739845.

51. Molyneaux R, Gibbs L, Bryant RA, Humphreys C, Hegarty K, Kellett C, et al. Interpersonal violence and mental health outcomes following disaster. BJPsych Open [Internet]. 2020 Jan [cited 2022 Jan 11];6(1). Available from: https://www.cambridge.org/core/journals/bjpsych-open/article/interpersonal-violence-and-mental-health-outcomes-following-disaster/FE519D59C68BCE944AE4A88884A157C5

52. Rao S. A natural disaster and intimate partner violence: Evidence over time. Social Science & Medicine. 2020 Feb 1;247:112804.

53. Devries KM, Mak JY, Bacchus LJ, Child JC, Falder G, Petzold M, et al. Intimate Partner Violence and Incident Depressive Symptoms and Suicide Attempts: A Systematic Review of Longitudinal Studies. PLoS Med. 2013 May 7;10(5):e1001439.

54. Machisa MT, Christofides N, Jewkes R. Mental ill health in structural pathways to women's experiences of intimate partner violence. PLOS ONE. 2017 Apr 6;12(4):e0175240.

55. Farmer P. An Anthropology of Structural Violence. Current Anthropology. 2004 Jun 1;45(3):305–25.

56. Finkelhor D, Ormrod RK, Turner HA. Polyvictimization and trauma in a national longitudinal cohort. Development and Psychopathology. 2007 Jan;19(1):149–66.

For peer review only

57. Herrenkohl TI, Sousa C, Tajima EA, Herrenkohl RC, Moylan CA. Intersection of child abuse and children's exposure to domestic violence. Trauma, violence & abuse. 2008 Apr;9(2):84–99.

58. Logie CH, Okumu M, Mwima S, Hakiza R, Irungi KP, Kyambadde P, et al. Social ecological factors associated with experiencing violence among urban refugee and displaced adolescent girls and young women in informal settlements in Kampala, Uganda: a cross-sectional study. Confl Health. 2019 Dec 17;13(1):60.

59. Jewkes R, Jordaan E, Myrttinen H, Gibbs A. Masculinities and violence: using latent class analysis to investigate the origins and correlates of differences between men in the cross-sectional UN Multi-country Study on men and violence in Asia and the Pacific. J Glob Health. 10(2):020439.

For peer review only

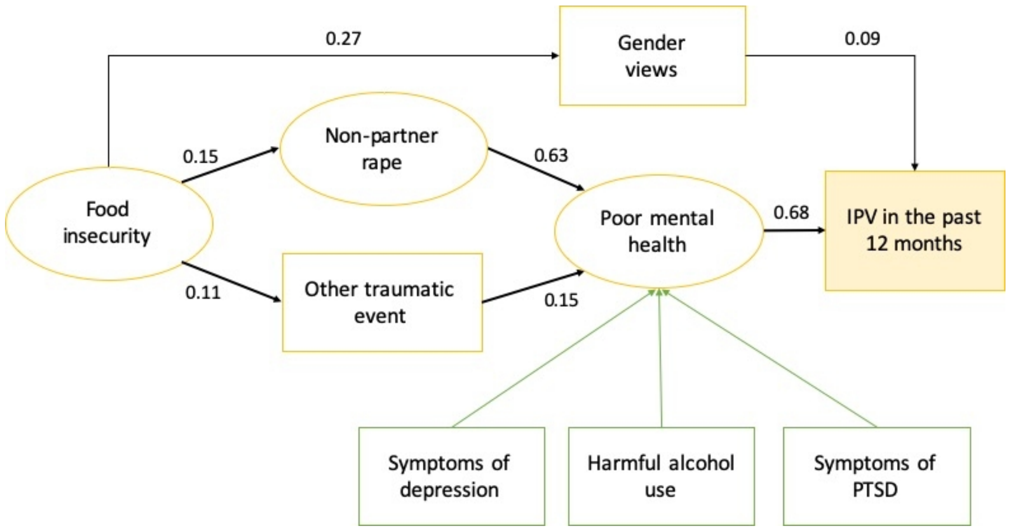

Structural equation model of pathways between exposure to traumatic events and IPV in the past 12 months (standardized coefficients)

277x144mm (72 x 72 DPI)

Reporting checklist for cross sectional study.

Based on the STROBE cross sectional guidelines.

Instructions to authors

Complete this checklist by entering the page numbers from your manuscript where readers will find each of the items listed below.

Your article may not currently address all the items on the checklist. Please modify your text to include the missing information. If you are certain that an item does not apply, please write "n/a" and provide a short explanation.

Upload your completed checklist as an extra file when you submit to a journal.

In your methods section, say that you used the STROBE cross sectionalreporting guidelines, and cite them as:

von Elm E, Altman DG, Egger M, Pocock SJ, Gotzsche PC, Vandenbroucke JP. The Strengthening the Reporting of Observational Studies in Epidemiology (STROBE) Statement: guidelines for reporting observational studies.

		Reporting Item	Page Number
**Title and abstract**			
Title	#1a	Indicate the study's design with a commonly used term in the title or the abstract	Page 1
Abstract	#1b	Provide in the abstract an informative and balanced summary of what was done and what was found	Page 2
**Introduction**			
Background / rationale	#2	Explain the scientific background and rationale for the investigation being reported	Page 5-6
Objectives	#3	State specific objectives, including any prespecified hypotheses	Page 6
**Methods**			
Study design	#4	Present key elements of study design early in the paper	Page 7

For peer review only

Setting	#5	Describe the setting, locations, and relevant dates, including periods of recruitment, exposure, follow-up, and data collection	Page 7
Eligibility criteria	#6a	Give the eligibility criteria, and the sources and methods of selection of participants.	Page 7
	#7	Clearly define all outcomes, exposures, predictors, potential confounders, and effect modifiers. Give diagnostic criteria, if applicable	Page 7
Data sources / measurement	#8	For each variable of interest give sources of data and details of methods of assessment (measurement). Describe comparability of assessment methods if there is more than one group. Give information separately for for exposed and unexposed groups if applicable.	7-9
Bias	#9	Describe any efforts to address potential sources of bias	7, 17
Study size	#10	Explain how the study size was arrived at	7
Quantitative variables	#11	Explain how quantitative variables were handled in the analyses. If applicable, describe which groupings were chosen, and why	7-9
Statistical methods	#12a	Describe all statistical methods, including those used to control for confounding	9-11
Statistical methods	#12b	Describe any methods used to examine subgroups and interactions	9-11
Statistical methods	#12c	Explain how missing data were addressed	NA
Statistical methods	#12d	If applicable, describe analytical methods taking account of sampling strategy	NA
Statistical methods	#12e	Describe any sensitivity analyses	9-11

Results

Participants	#13a	Report numbers of individuals at each stage of study—eg numbers potentially eligible, examined for eligibility, confirmed eligible, included in the study, completing follow-up, and analysed. Give information separately for for exposed and unexposed groups if applicable.	NA

For peer review only

Participants	#13b	Give reasons for non-participation at each stage	NA
Participants	#13c	Consider use of a flow diagram	NA
Descriptive data	#14a	Give characteristics of study participants (eg demographic, clinical, social) and information on exposures and potential confounders. Give information separately for exposed and unexposed groups if applicable.	11-12
Descriptive data	#14b	Indicate number of participants with missing data for each variable of interest	NA
Outcome data	#15	Report numbers of outcome events or summary measures. Give information separately for exposed and unexposed groups if applicable.	11-13
Main results	#16a	Give unadjusted estimates and, if applicable, confounder-adjusted estimates and their precision (eg, 95% confidence interval). Make clear which confounders were adjusted for and why they were included	14
Main results	#16b	Report category boundaries when continuous variables were categorized	7-9
Main results	#16c	If relevant, consider translating estimates of relative risk into absolute risk for a meaningful time period	NA
Other analyses	#17	Report other analyses done—e.g., analyses of subgroups and interactions, and sensitivity analyses	14-15
**Discussion**			
Key results	#18	Summarise key results with reference to study objectives	15-17
Limitations	#19	Discuss limitations of the study, taking into account sources of potential bias or imprecision. Discuss both direction and magnitude of any potential bias.	17
Interpretation	#20	Give a cautious overall interpretation considering objectives, limitations, multiplicity of analyses, results from similar studies, and other relevant evidence.	15-18
Generalisability	#21	Discuss the generalisability (external validity) of the study results	17

For peer review only

Other Information

Funding	#22	Give the source of funding and the role of the funders for the present study and, if applicable, for the original study on which the present article is based	19

None The STROBE checklist is distributed under the terms of the Creative Commons Attribution License CC-BY. This checklist can be completed online using https://www.goodreports.org/, a tool made by the EQUATOR Network in collaboration with Penelope.ai

For peer review only
